# HOMER3 facilitates growth factor-mediated β-Catenin tyrosine phosphorylation and activation to promote metastasis in triple negative breast cancer

**DOI:** 10.1186/s13045-020-01021-x

**Published:** 2021-01-06

**Authors:** Qinghua Liu, Lixin He, Siqi Li, Fengyan Li, Guangzheng Deng, Xinjian Huang, Muwen Yang, Yunyun Xiao, Xiangfu Chen, Ying Ouyang, Jinxin Chen, Xuxia Wu, Xi Wang, Libing Song, Chuyong Lin

**Affiliations:** 1 State Key Laboratory of Oncology in Southern China and Department of Experimental Research, Collaborative Innovation Center for Cancer Medicine, Sun Yat-sen University Cancer Center, Guangzhou, 510060 China; 2 State Key Laboratory of Ophthalmology, Zhongshan Ophthalmic Center, Sun Yat-sen University, Guangzhou, 510060 China; 3 Department of Breast Surgery, Cancer Center, Sun Yat-sen University, Guangzhou, 510060 China; 4 Department of Radiation Oncology, Cancer Center, Sun Yat-sen University, Guangzhou, 510060 China

**Keywords:** HOMER3, β-catenin, Tyrosine phosphorylation, Metastasis, TNBC

## Abstract

**Background:**

HOMER family scaffolding proteins (HOMER1-3) play critical roles in the development and progression of human disease by regulating the assembly of signal transduction complexes in response to extrinsic stimuli. However, the role of HOMER protein in breast cancer remains unclear.

**Methods:**

HOMER3 expression was examined by immunohistochemistry in breast cancer patient specimens, and its significance in prognosis was assessed by Kaplan–Meier survival analysis. The effects of HOMER3 in growth factor-induced β-Catenin activation were analyzed by assays such as TOP/FOP flash reporter, tyrosine phosphorylation assay and reciprocal immunoprecipitation (IP) assay. Role of HOMER3 in breast cancer metastasis was determined by cell function assays and mice tumor models.

**Results:**

Herein, we find that, among the three HOMER proteins, HOMER3 is selectively overexpressed in the most aggressive triple negative breast cancer (TNBC) subtype, and significantly correlates with earlier tumor metastasis and shorter patient survival. Mechanismly, HOMER3 interacts with both c-Src and β-Catenin, thus providing a scaffolding platform to facilitate c-Src-induced β-Catenin tyrosine phosphorylation under growth factor stimulation. HOMER3 promotes β-Catenin nuclear translocation and activation, and this axis is clinically relevant. HOMER3 promotes and is essential for EGF-induced aggressiveness and metastasis of TNBC cells both in vitro and in vivo.

**Conclusion:**

These findings identify a novel role of HOMER3 in the transduction of growth factor-mediated β-Catenin activation and suggest that HOMER3 might be a targetable vulnerability of TNBC.

## Introduction

Breast cancer incidence has increased steadily during the past decades and is currently the most common malignancies in females worldwide [1]. There are four major subtypes of breast cancer: Luminal A, Luminal B, HER2 and Triple-negative breast cancer (TNBC). TNBC, defined by lack of clinicopathological expression of hormonal receptors and Her2, is the most aggressive subtype that accounts for only 15% of incidence but approximate 30% of breast cancer-related deaths [2, 3]. TNBC could not be treated with endocrine or targeted therapy [4]. Despite recent advances in chemotherapy, TNBC treatments are either transient or effective in a limited set of patients, and can be associated with severe toxicity [5]. Moreover, TNBC cells are characterized by exceeding mitotic, invasive, migratory features for distant dissemination, leading to frequent tumor recurrence [6]. Thus, the field is in need of new biomarkers and therapeutic targets.

β-Catenin, central effector of Wnt signaling pathway, mainly serves as a transcriptional co-activator of T-cell factors/lymphoid enhancer factor (TCFs/LEF) to dictate downstream genes transcription, and plays crucial roles in development and progression of human diseases [7]. Unsurprisingly, β-Catenin activity is enriched in BLBC and predicts poor prognosis of TNBC patients [8, 9]. Studies have demonstrated aberrant activation of β-Catenin promotes cell migration, invasion and stem cell-like properties of TNBC which encourage metastasis [10]. However, mutations of key components of Wnt/β-Catenin signaling pathway such as APC and β-Catenin that have been proved to contribute to colorectal cancer, and other malignancies are not involved in the enrichment of β-Catenin signal in TNBC [11].

Crosstalk between growth-factor signal transduction and canonical Wnt pathway allows β-Catenin activation in Wnt-independent ways. As a common joint of multiple signaling pathways, β-Catenin can be phosphorylated on tyrosine residues upon the effective stimulation of growth factors, resulting in excessive β-Catenin nuclear translocation and transcriptional activation of targeted genes in the context of malignant disease [12–14]. Nevertheless, whether the aggressive traits of TNBC cells could be attributed to the tyrosine phosphorylation of β-Catenin remain largely elusive.

HOMER family scaffolding proteins comprise three members (HOMER1-3) in mammals [15, 16]. Whereas the carboxyl-terminal Coiled-coil self-assembly domain allows HOMER proteins to build multimeric platform for scaffolding function, the enabled/vasodilator-stimulated phosphoprotein homology 1 (EVH1) domain located in amino-terminus is responsible for the recognition of different functional molecules such as phosphoinositide 3-kinase enhancer long isoform (PIKE-L), group 1 metabotropic glutamate receptors (mGluRs), IP3 receptors (IP3R) and Shanks to participate in intracellular calcium release and glutamatergic signaling transduction [16–21]. These HOMER proteins predominately expressed in the nervous system, peripheral tissues, and particularly in human cancers to regulate cell growth, migration and apoptosis [20, 22–25]. These pieces of evidence suggest that HOMER proteins may play important roles in carcinogenesis by affecting oncogenic pathways. However, the roles of HOMER family in human breast cancer, especially in TNBC, are still unclear.

In the present study, we find that, among the HOMER family members, HOMER3 is selectively overexpressed in TNBC and correlates with poor prognosis. Notably, HOMER3 plays a scaffolding function to simultaneously interact with c-Src and β-Catenin and promotes their efficient interactions, thus facilitating the growth factor-induced β-Catenin tyrosine phosphorylation and activation. HOMER3 is essential for EGF-mediated aggressiveness and metastasis both in vitro and in vivo. These findings uncover the role of HOMER3 in growth-factor-induced β-Catenin activation and TNBC metastasis, and open new avenues to prevent or overcome TNBC by targeting HOMER3.

## Materials and methods

### Cells

Breast cancer cell lines were obtained from the ATCC cultured following the provider’s recommendations. MCF-7, T47D, MDA-MB-231 were grown in DMEM medium supplemented with 10% fetal bovine serum (FBS). ZR-75–1, BT-549 and 4T1 cells were maintained in RPMI-1640 medium with 10% FBS. SUM159PT cells were maintained in Ham's F-12 with 10% FBS. All cells were supplemented with penicillin/streptomycin, hydrocortisone, insulin, HEPES and L-glutamine, and maintained at 37 °C in 5% CO2. Cell lines were authenticated by short tandem repeat (STR) fingerprinting.

### Patient information and tissue specimens

This study was conducted on a total of 347 paraffin-embedded breast cancer samples including 256 non-TNBCs and 91 TNBCs, which had been histopathologically and clinically diagnosed at the Sun Yat-sen University Cancer Center from 2005 to 2013. Clinicopathological characteristics are summarized in Additional file 7: Table 1. Ethics approval and prior patient consent had been obtained from the Institutional Research Ethics Committee for the use of the clinical specimens for research purposes.

### Immunohistochemistry (IHC)

IHC staining was performed on the 347 paraffin-embedded breast cancer tissue sections using HOMER3 and β-Catenin antibodies as previously reported [26]. The results of the staining were evaluated and scored by two independent pathologists, who were blinded to the clinical outcome. The HOMER3 staining was graded with four scores, strong + 3, moderate + 2, weak + 1, and negative 0. Specimens with scores + 3, + 2 were defined as high expression; while the others scored as + 1 or 0 were low expression. On the other hand, specimens with > 10% nuclear β-Catenin expression were defined as nuclear β-Catenin-positive, and specimens with ≤ 10% nuclear β-Catenin expression were nuclear β-Catenin-negative. Details of the IHC method were provided in Additional file 7.

### Xenograft tumor models

Breast cancer spontaneous metastasis model and lung colonization model were performed as previously described [26]. Female BALB/c or BALB/c-nude mice (5–6 weeks old) were purchased and housed in barrier facilities on a 12-h light/dark cycle. The Institutional Animal Care and Use Committee of Sun Yat-sen University approved all experimental procedures. For spontaneous metastasis assays, 4T1-luciferase cells (2 × 10^5^) with or without HOMER3 knockdown were orthotopically injected into the mammary fat pads of BALB/c mice and inoculated for 5 weeks. Metastasis was detected using the IVIS imagining system (Caliper) by blocking the orthotopic tumor signals.

In the lung colonization model, BALB/c-nude mice were intravenously injected with control or HOMER3 silencing MDA-MB-231-luc cells (1 × 10^6^, *n* = 8/group). Lung metastasis burden of animals was monitored weekly using bioluminescent imaging (BLI). Mice were euthanized 9 weeks after inoculation, and lung metastases were evaluated.

In the in vivo experiments, mice were subcutaneously administered with EGF (1 mg/kg, mEGF for 4T1 and hEGF for MDA-MB-231) every other day as previously reported [27]. For validation, lungs were fixed in formalin and embedded in paraffin using a routine method and subjected for H&E staining. Lung surface metastatic lesions were counted under a dissecting microscope and presented as the mean ± s.e.m..

### Immunoprecipitation (IP) assays

Cell lysates were prepared from the indicated cells using lysis buffer (150 mM NaCl, 10 mM HEPES, pH 7.4, 1% NP-40). Lysates were then incubated with anti-HOMER3 (Sigma-Aldrich, rabbit mAb), or anti-c-Src, or anti-β-Catenin (Cell Signaling Technology, mouse or rabbit mAb) antibody, and protein G-conjugated agarose, or Flag, Myc, HA affinity agarose (Sigma-Aldrich), at 4 °C overnight. Beads containing affinity-bound proteins were washed 6 times by IP wash buffer (150 mM NaCl, 10 mM HEPES, pH 7.4, 0.1% NP-40), followed by eluting using 1 M glycine (pH 3.0). The eluates were then mixed with sample buffer and denatured and used for the western blot analysis. Target proteins were blot with primary antibodies derived from biological sources different from those used in IP to avoid high background. For the detection of β-Catenin Tyr phosphorylation levels, cell lysates were first pulldown with anti-β-Catenin antibody (Cell Signaling Technology, mouse mAb) and subjected for western blot, followed by staining with p-Tyr specific antibody (Cell Signaling Technology, rabbit mAb).

Protein purification was performed as previously reported [28]. Briefly, 5 × 10^7^ 293 T cells transfected with 200 μg Flag-HOMER3, or Flag-c-Src, or Flag-β-Catenin expressing plasmid was lysed using RIPA buffer (0.25% SDS). Lysates were then incubated with 200-μl Flag affinity agarose (Sigma-Aldrich) overnight at 4 °C. Beads containing affinity-bound proteins were washed six times by 5-ml RIPA buffer, followed by elution with 500 μl of Flag competing peptides (Sigma-Aldrich, 0.1 μg/μl) twice. The elutes were pooled and washed with 5 ml PBS using 3-kDa MW cutoff filter units (Millipore) to remove the competing peptides. The purities of HOMER3, c-Src and β-Catenin were examined by SDS-PAGE and Coomassie blue staining and then subjected for in vitro binding assays.

### Cytokines and cell treatment

Recombinant proteins including Wnt3A, EGF and TGFα were purchased from R&D system (Minneapolis, MN, USA) and SinoBiological (Beijing, China). Before treatment, cells were changed to 1% FBS medium culturing for 1 h; cytokines were then added to a final concentration of 20 ng/ml for Wnt3A, 20 ng/ml for EGF, and 10 ng/ml for TGFα, respectively. For the matrigel 3-D spheroid formation assay, cells were replenished with 50-μl serum-free or EGF-supplemented medium every other day.

### TOP/FOP flash activity assays

The wild-type (TOP) and mutant (FOP) LEF/TCF reporters were cloned into pGL3 luciferase constructs (Promega). Twenty thousand cells were seeded in triplicate in 48-well plates and allowed to settle for 24 h. One hundred nanograms of TOP or FOP flash, plus 1 ng of pRL-TK Renilla plasmid (Promega), was transfected into cells using the Lipofectamine 3000 reagent according to the manufacturer's recommendation. Luciferase and Renilla signals were measured 24 h after transfection using the Dual Luciferase Reporter Assay Kit (Promega) according to a protocol provided by the manufacturer. The results were calculated as the ratio of specific TOP-Flash over non-specific FOP-Flash relative renilla luciferase units (RLU).

### Statistical analysis

Statistical analyses were performed using the SPSS version 19.0 statistical software package. Statistical tests for data analysis included the log-rank test, χ^2^ test, Spearman-rank correlation test and Student’s t test (two-tailed). Multivariate statistical analysis was performed using a Cox regression model. *P* < 0.05 was considered statistically significant.

Additional information is provided in Supplementary Materials and Methods.

## Result

### HOMER3 is substantially increased in TNBC and correlates with poor prognosis

HOMER scaffolding proteins mediate cellular signaling transduction and emerge as important regulators in development and disease [15, 16]. To investigate the potential roles of HOMER genes in breast cancer, we first explored the expression patterns of HOMER1-3 in the Cancer Genome Atlas (TCGA) breast cancer datasets (BRCA). Intriguingly, HOMER3, but not HOMER1 or HOMER2, HOMER3 showed a step-wise increase toward aggressiveness, indicating the highest expression in TNBC or Basal-like tumors which share a ~ 80% overlap with TNBC [29] (Additional file 1: Fig. 1a, b). Similarly, our real-time PCR analysis revealed that only HOMER3 was robustly elevated in TNBC compared to non-TNBC and normal breast tissues (Additional file 1: Fig. 1C). We then focused on the role of HOMER3 in breast cancer.

**Fig. 1:**
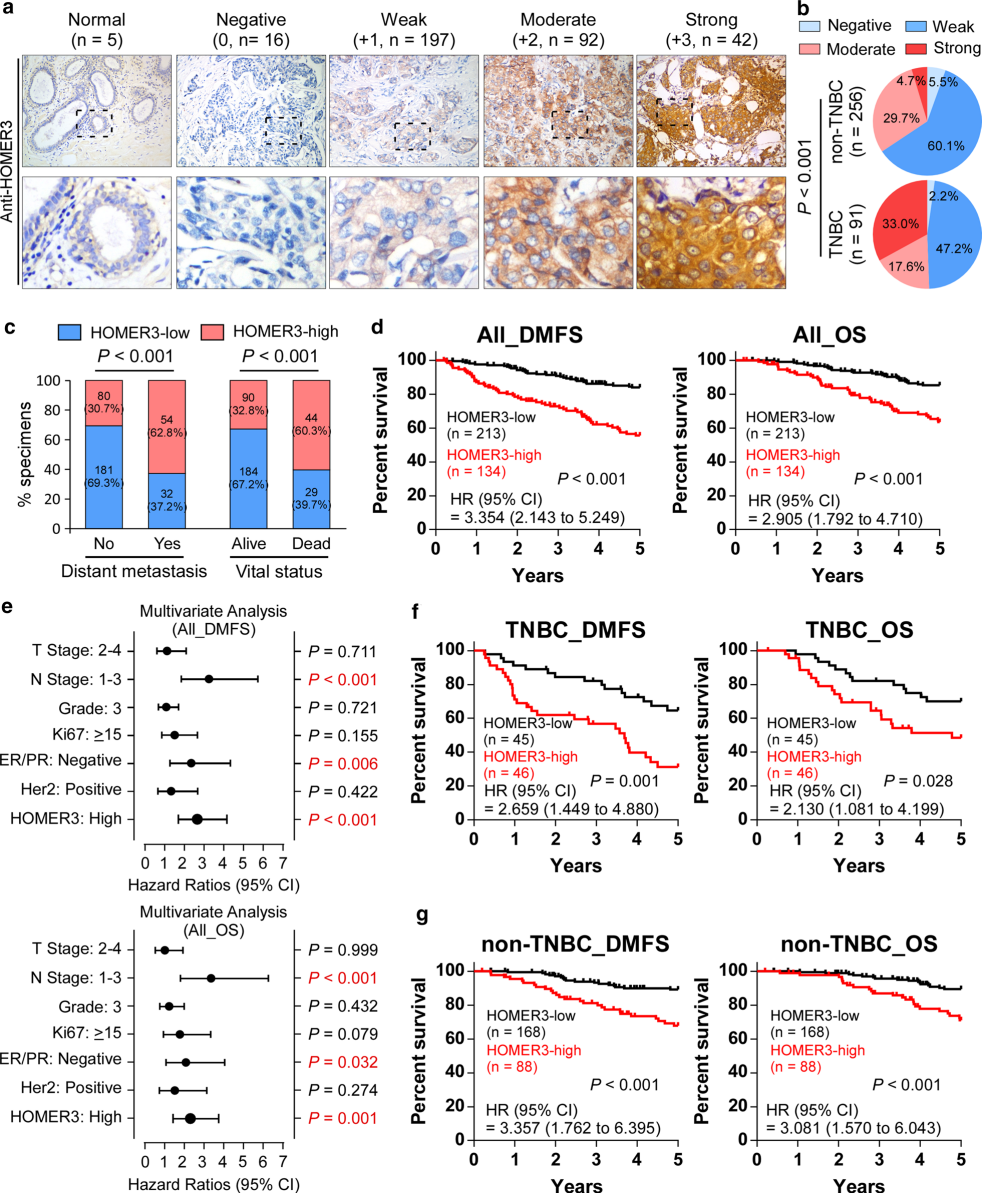
HOMER3 is substantially increased in TNBC and correlates with poor prognosis. **a** Representative images of HOMER3 IHC staining in 5 normal breast tissues, 91 TNBC and 256 non-TNBC specimens, scored as negative (0), weak (+ 1), moderate (+ 2) or strong (+ 3). Number with indicated scores was shown in brackets. **b** The distribution of HOMER3 staining in TNBC and non-TNBC patient specimens was shown and compared. χ^2^ test was used. **c** HOMER3 significantly correlated with the status of tumor metastasis and patient survival. χ^2^ test was used. **d** Kaplan–Meier analysis of 5-year distant metastasis-free survival (DMFS) and overall survival (OS) in breast cancer patients stratified by low and high HOMER3 expression (*n* = 347, log-rank test). HR, hazard ratio. **e** Multivariate Cox regression analysis to evaluate the significance of the association between HOMER3 expression signature and 5-year DMFS and OS in the presence of other important clinical variables. **f, g** High levels of HOMER3 significantly predicted poorer 5-year DMFS and OS in both TNBC (**f**) and non-TNBC (**g**) subgroups

The clinical significance of HOMER3 expression was further assessed in the 347 breast cancer specimens (Additional file 7: Table S1). Consistently, we found a significant increase in HOMER3 expression in TNBC patient specimens (Fig. 1a, b). Correlation analysis showed that high levels of HOMER3 were significantly associated with advanced clinical stage, lymph node metastasis, tumor size, as well as 5-year distant metastasis status and patient vital status (Fig. 1c and Additional file 7: Table S2). Importantly, breast cancer patients with high HOMER3 expression significantly suffered earlier metastasis and shorter survival time (Fig. 1d), and this high HOMER3 expression signature could be recognized as one of the independent prognostic factors for the prognosis of tumor metastasis and patient survival (Fig. 1e). Notably, despite a differential distribution, high HOMER3 elicited significant role in predicting poorer DMFS and OS in both TNBC and non-TNBC, suggesting that high HOMER3 may contribute to malignant progression of both subtypes (Fig. 1f, g). In addition, analyses with Kaplan–Meier Plotter program (http://kmplot.com/analysis) indicated that high expression of HOMER3 was significantly associated with tumor metastasis, relapse and patient survival in breast cancer (Additional file 1: Fig. 1D). Collectively, these results reveal that robust increase of HOMER3 may play an important role in the progression of breast cancer, especially in TNBC.

### HOMER3 promotes growth factor-induced β-Catenin tyrosine phosphorylation and activation

**Fig. 2:**
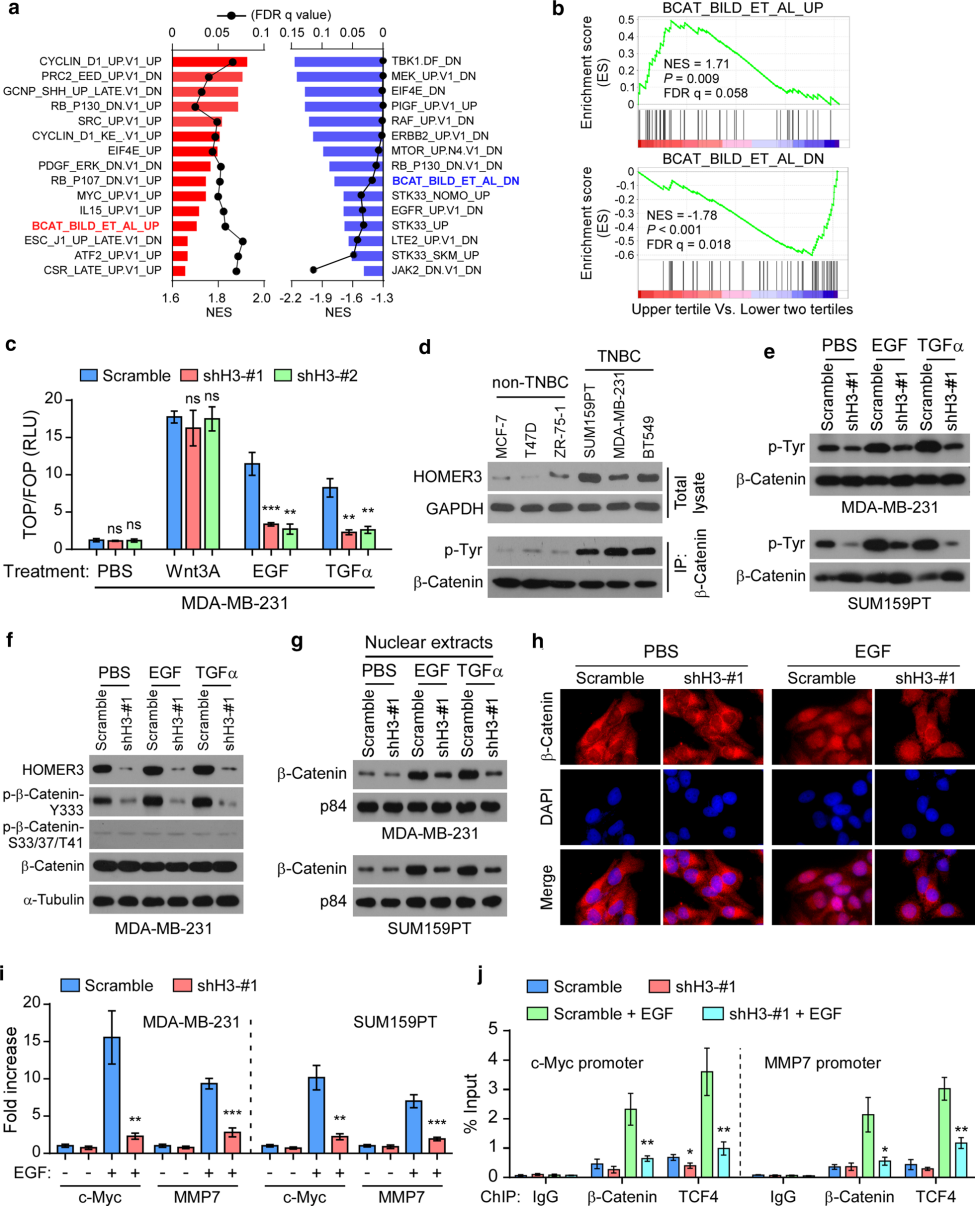
HOMER3 promotes growth factor-induced β-Catenin tyrosine phosphorylation and activation. **a** Gene set enrichment analysis (GSEA) of TCGA breast cancer stratified by HOMER3 expression (upper tertile vs. lower two tertiles) using the C6 signature set (oncogenic signatures). The most 15 significantly correlated signatures (positively and negatively) were shown. FDR q, false discovery rate q value; NES, normalized enrichment score. **b** Enrichment of β-Catenin-upregulated gene signature (BCAT_BILD_ET_AL_UP) and β-Catenin-repressed gene signature (BCAT_BILD_ET_AL_DN) was indicated. **c** Normalized luciferase activities of specific TOP-Flash over non-specific FOP-Flash relative renilla luciferase units (RLU) in MDA-MB-231 cells treated with PBS, Wnt3A, EGF or TGFα. ns, not significant. **d** Endogenous levels of HOMER3 in non-TNBC and TNBC cell lines. For tyrosine phosphorylation examination, cell lysates from indicated cell lines were subjected for immunoprecipitation of β-Catenin, followed by western blot analysis with p-Tyr-specific antibody and total β-Catenin antibody. **e** Tyr phosphorylation levels of β-Catenin in control and HOMER3 silencing MDA-MB-231 and SUM159PT cells treated with PBS, EGF or TGFα. **f** Western blot analysis of HOMER3, p-β-Catenin-Y333, p-β-Catenin-S33/37/T41 and total β-Catenin in indicated MDA-MB-231 cells. **g** Nuclear fractions were extracted and subjected for examination of β-Catenin protein. P84 was used as nuclear loading control. **h** Fluorescence immunostaining of β-Catenin expression in control and HOMER3 silencing MDA-MB-231 cells with PBS or EGF treatment. **i** Relative expression levels of two typical β-Catenin downstream genes c-Myc and MMP-7 were examined by real-time PCR. **j** Enrichment of β-Catenin and TCF-4 on c-Myc and MMP-7 promoters was determined by ChIP assays. *P < 0.05; **P < 0.01; ***P < 0.001

Considering that HOMER3 is a scaffolding protein, we then performed a Gene set enrichment analysis (GSEA) of the TCGA breast cancer samples according to HOMER3 expression to identify its regulated oncogenic signals. Interestingly, the GSEA analysis showed consistent enrichment of β-Catenin-upregulated gene signature (BCAT_BILD_ET_AL_UP) in HOMER3-high subgroup, and β-Catenin-repressed gene signature (BCAT_BILD_ET_AL_DN) in HOMER3-low samples, suggesting that HOMER3 may regulate the β-Catenin activity (Fig. 2a, b).

In fact, β-Catenin can be activated by cytokines including the Wnt family proteins and growth factors [12–14]. Surprisingly, HOMER3 silencing strikingly repressed EGF- and TGF-α-induced TOP/FOP Flash activities, but had no significant effects on Wnt3A-mediated β-Catenin activation (Fig. 2c). Moreover, whereas silencing of HOMER3 significantly inhibited TOP/FOP Flash activities in MDA-MB-231 and SUM159PT cells cultured with 10% FBS, the activities of TOP/FOP Flash remained at relative low levels and were not significantly affected by HOMER3 shRNAs cultured with limited 1% FBS (Additional file 2: Fig. 2A). These findings suggest that HOMER3 may promote the signal transduction of growth factor-induced β-Catenin activation.

Growth factors increase tyrosine phosphorylation of β-Catenin to promote its nuclear translocation and transcriptional activation [12, 13]. Indeed, EGFR and c-Src are expressed and could be activated by EGF, suggesting that the EGFR/c-Src signaling is intact in breast cancer cell lines (Additional file 2: Fig. 2B). As expected, the levels of tyrosine (Tyr) phosphorylation of β-Catenin were strikingly increased in TNBC cell lines that had high HOMER3 expression (Fig. 2d). Although HOMER3 did not regulate the phosphorylation of EGFR or c-Src, silencing of HOMER3 robustly abrogated EGF- and TGF-α-induced Tyr phosphorylation of β-Catenin in TNBC MDA-MB-231 and SUM159PT cells (Fig. 2e and Additional file 2: Fig. 2C). Moreover, when referred to a commercial anti-p-β-Catenin-Y333 antibody, similar repression of β-Catenin Tyr phosphorylation by HOMER3 silencing was also observed. In contrast, downregulation of HOMER3 had subtle effects on levels of total and serine/threonine phosphorylation of β-Catenin (Fig. 2f). Although HOMER3 expression was not regulated by EGF or TGF-α treatment (Fig. 2f and Additional file 2: Fig. 2D), silencing of HOMER3 abrogated EGF-mediated nuclear translocation of β-Catenin in MDA-MB-231 and SUM159PT cells (Fig. 2g, h). Moreover, downregulation of HOMER3 significantly repressed the expression of two typical β-Catenin downstream genes c-Myc and MMP-7, as well as the enrichment of β-Catenin and TCF4 on their promoters (Fig. 2i, j). These findings indicate that HOMER3 promotes growth factor-induced β-Catenin tyrosine phosphorylation and activation.

### HOMER3 interacts with tyrosine-protein kinase c-Src

**Fig. 3:**
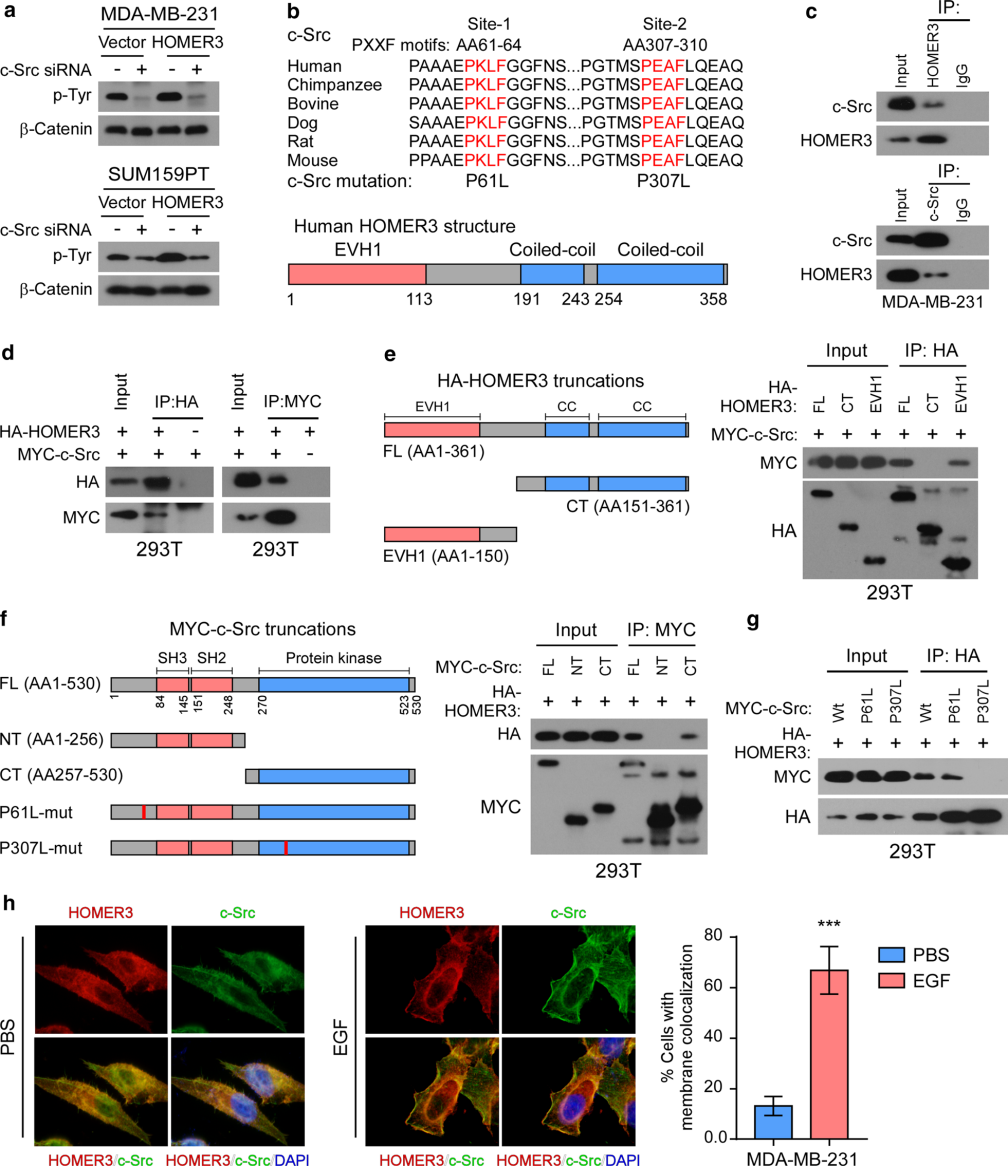
HOMER3 interacts with tyrosine-protein kinase c-Src. **a** Tyr phosphorylation levels of β-Catenin were examined in control and HOMER3-overexpressing MDA-MB-231 and SUM159PT cells with or without c-Src knockdown. **b** Schematic illustration of two conserved PxxF (x, any amino acid) motifs in c-Src protein that might be recognized by the EVH1 domain of HOMER3. **c** Immunoprecipitation (IP) assays determined the interaction between endogenous HOMER3 and c-Src in MDA-MB-231 cells. **d** IP assays were performed in 293 T cells transfected with HA-HOMER3 and MYC-c-Src constructs. **e** Left panel: Schematic illustration of HOMER3 truncated constructs. Right panel: The 293 T cells were transfected with indicated HA-tagged HOMER3 truncations and MYC-c-Src, followed by IP assays with HA-beads to examine their interaction with c-Src. **f** Left panel: Sketch of MYC-c-Src truncations. Right panel: The 293 T cells were transfected with indicated MYC-c-Src truncations and HA-HOMER3, followed by IP assays with MYC-beads to examine their interaction with HOMER3. **g** IP assays examined the interactions between HOMER3 and wild-type (wt) or mutant (P61L and P307L) c-Src proteins. **h** MDA-MB-231 cells were first stimulated with EGF for a short time, and then fluorescence immunostaining of HOMER3 and c-Src was performed to examine their colonization. Statistical analysis indicated the percentage of cells that showed membrane colonization of c-Src and HOMER3. ***P < 0.001

Previous studies have identified c-Src as a direct upstream tyrosine-protein kinase of β-Catenin [12, 30]. Notably, silencing of c-Src abrogated HOMER3-induced Tyr phosphorylation and activation of β-Catenin in TNBC cells (Fig. 3a and Additional file 3: Fig. 3A-B), suggesting that c-Src was responsible for the function of HOMER3 in β-Catenin phosphorylation. Interestingly, analysis of the c-Src peptide sequence showed two conserved PXXF motifs that could be recognized by the EVH1 domain of HOMER3 [20] (Fig. 3b). Strikingly, endogenous and exogenous reciprocal immunoprecipitation (IP) assays revealed that HOMER3 interacted with c-Src (Fig. 3c, d).

To further understand the details of the interactions between HOMER3 and c-Src, truncated forms of HOMER3 and c-Src were expressed and subjected for IP assays. We found that only those HOMER3 truncations with EVH1 domain could bind to c-Src, suggesting that this domain was responsible for its interaction with c-Src (Fig. 3e). On the other hand, the C-terminal protein kinase domain was essential for c-Src to interact with HOMER3 (Fig. 3f). Notably, the P307L, but not P61L, mutation of c-Src abrogated the interaction between HOMER3 and c-Src, indicating that the second PXXF motif was indispensable for the recognition of HOMER3 EVH1 domain (Fig. 3g). Moreover, immunofluorescence (IF) staining revealed that HOMER3 and c-Src colonized in cytoplasm and were both recruited to plasma membrane when stimulated with EGF (Fig. 3h). Thus, these results reveal that HOMER3 interacts with c-Src to facilitate Tyr phosphorylation of β-Catenin.

### HOMER3 functions as a scaffolding platform to facilitate c-Src and β-Catenin interaction

**Fig. 4:**
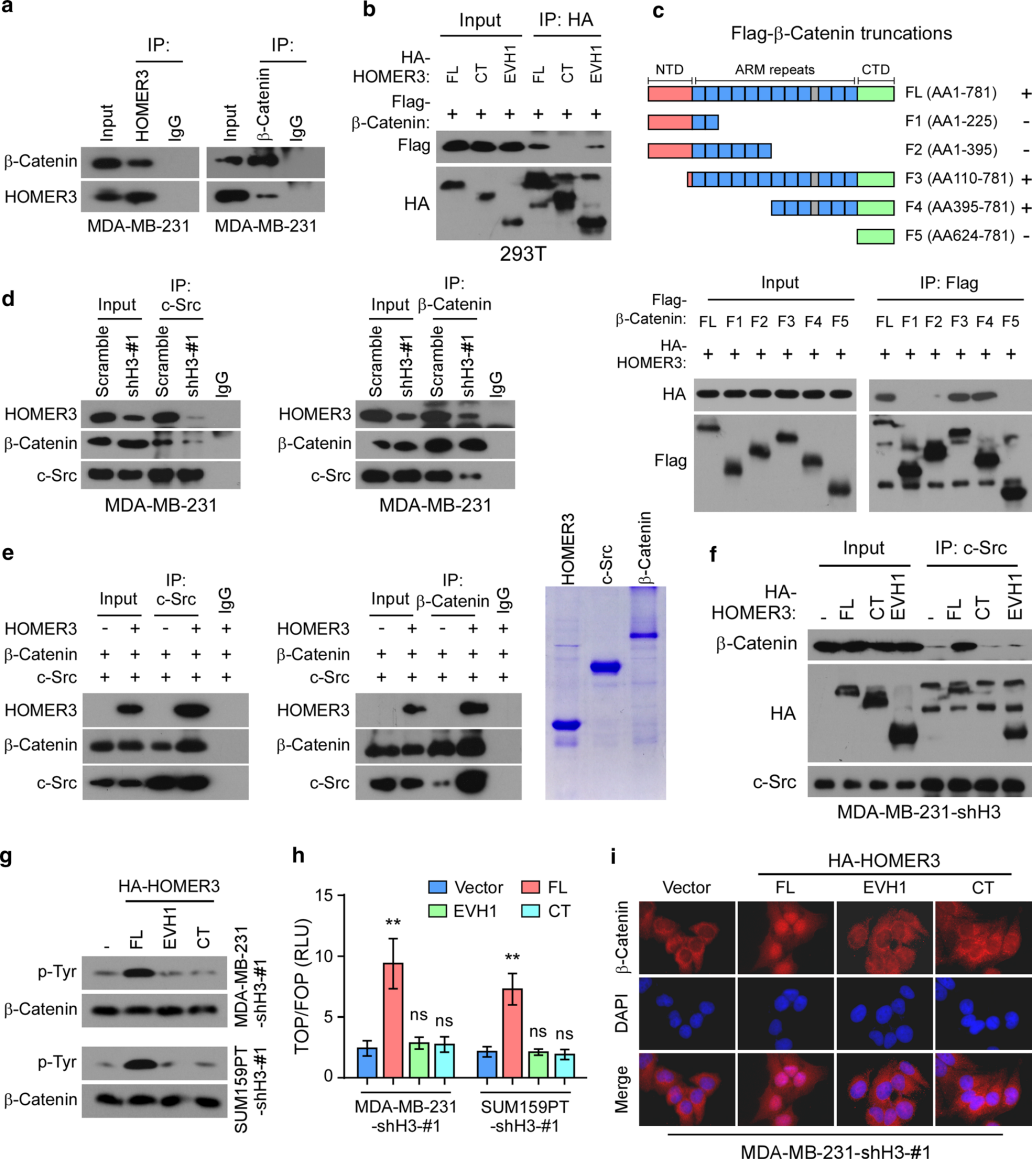
HOMER3 functions as a scaffold platform to facilitate c-Src and β-Catenin interaction. **a** The interaction between HOMER3 and β-Catenin was analyzed by IP assays. **b** Interactions between β-Catenin and truncated HOMER3 proteins. **c** Up panel: Schematic illustration of β-Catenin truncated constructs. Low panel: 293 T cells were transfected with indicated Flag-β-Catenin truncations and HA-HOMER3, followed by IP assays with Flag-beads to examine their interaction with HOMER3. **d** Reciprocal IP assays of c-Src and β-Catenin in MDA-MB-231 cells with or without HOMER3 silencing. **e** In vitro protein binding assays with purified HOMER3, β-Catenin and c-Src protein. The purities of HOMER3, c-Src and β-Catenin were examined by SDS-PAGE and Coomassie Blue Staining. **f** IP assays of c-Src in HOMER3 silencing MDA-MB-231 cells with restoration of full-length or truncated HOMER3 constructs. **g** Tyr phosphorylation of β-Catenin in HOMER3 silencing cells with restoration of full-length or truncated HOMER3 constructs. **g** Normalized luciferase activities of specific TOP-Flash over non-specific FOP-Flash relative renilla luciferase units (RLU) in indicated cells. **h** Fluorescence immunostaining of β-Catenin expression in HOMER3 silencing MDA-MB-231 cells with restoration of indicated HOMER3 constructs

We next investigate whether HOMER3 serves as a scaffolding protein where c-Src and β-Catenin could bind, and therefore shortens their spatial distance for more efficient interaction. The IP assays showed reciprocal interactions between HOMER3 and β-Catenin (Fig. 4a). Moreover, IP analysis of protein truncations indicated that the EVH1 domain of HOMER3, and the ARM repeats of β-Catenin are indispensable for interactions (Fig. 4b, c). Importantly, silencing of HOMER3 strikingly diminished the endogenous interactions between c-Src and β-Catenin (Fig. 4d). To further strengthen that HOMER3 could increase the efficiency of c-Src and β-Catenin interaction, we then purified these three proteins and performed the in vitro binding assays (Fig. 4e). Consistent with previous report [12], c-Src could directly bind β-Catenin. Strikingly, the interactions between c-Src and β-Catenin were substantially enhanced by adding the purified HOMER3 protein (Fig. 4e), suggesting that HOMER3 indeed facilitates c-Src and β-Catenin interaction.

Notably, HOMER proteins usually orchestrate homomeric platform through their coiled-coil domain (CC domain) for efficient interaction with substrates [16]. To further assess whether the CC domain-mediated homomeric status is required for its scaffolding function, we reintroduced HOMER3 full-length or truncated constructs into the HOMER3-silencing TNBC cells. Of note, overexpression of HOMER3 EVH1 or CC domain, which lacks the capacity to recognize substrate or form dimerization, failed to restore the interaction between c-Src and β-Catenin in HOMER3-silencing MDA-MB-231 cells, while reconstitution of full-length HOMER3 restored the interaction (Fig. 4f). Moreover, reconstitution of full-length HOMER3, but not the individual EVH1 or CC domain, was capable to restore the Tyr phosphorylation of β-Catenin, TOP/FOP activity and nuclear location of β-Catenin in HOMER3-silencing MDA-MB-231 and SUM159PT cells (Fig. 4g, i). These findings further indicate that both the EVH1 and CC domains are necessary, but not sufficient, for the complete function of HOMER3 in mediating the efficient interaction between c-Src and β-Catenin.

### HOMER3 is essential for EGF-mediated aggressiveness in breast cancer

**Fig. 5:**
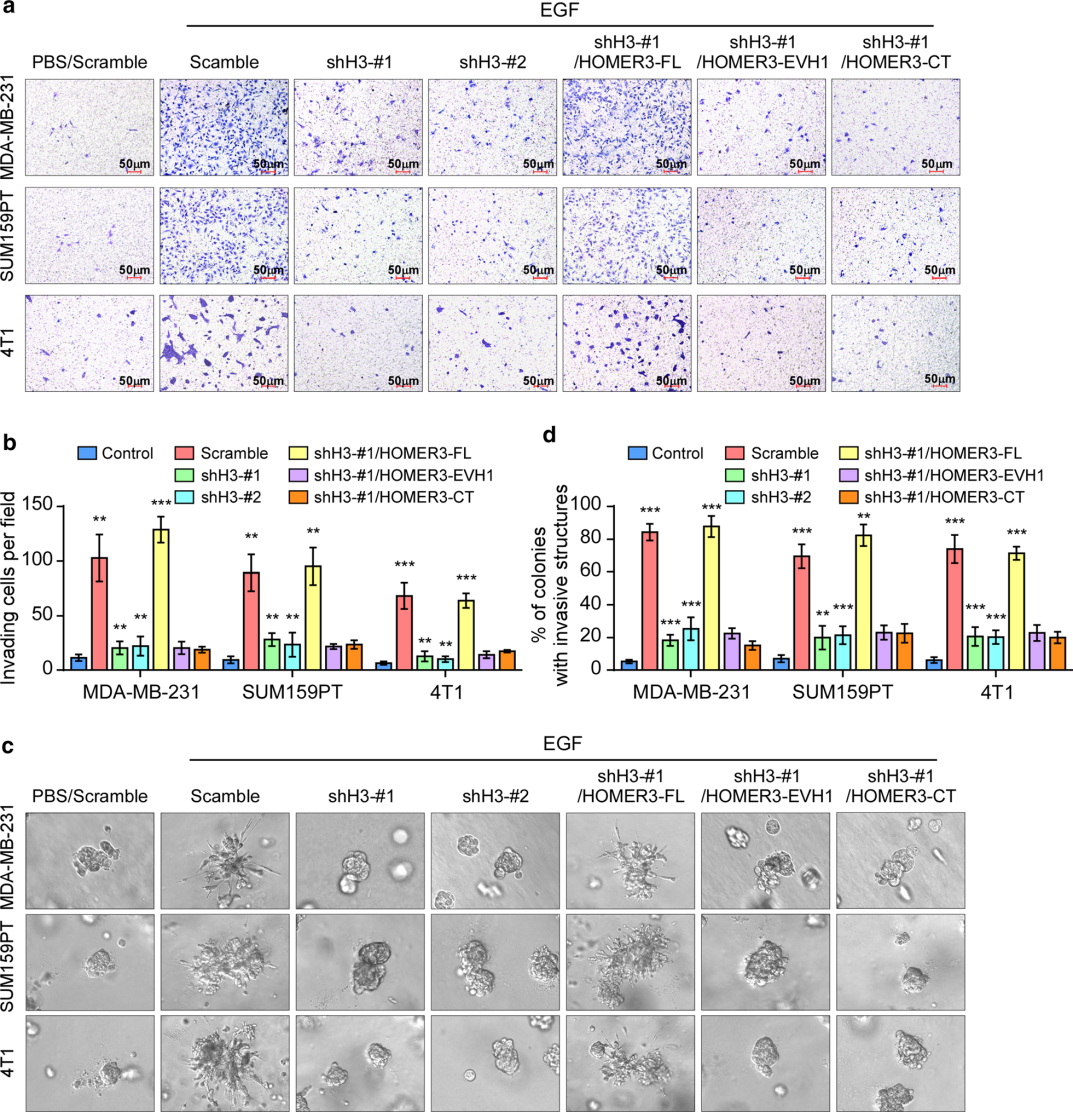
HOMER3 is essential for EGF-mediated aggressiveness in TNBC. **a, b** TNBC cells that had HOMER3 knockdown, or restoration of indicated HOMER3 truncated constructs, were treated with EGF, and subjected for transwell matrix penetration assays. Representative images (**a**) and quantification (**b**) of invading TNBC cells were shown. **c** 3-D spheroid cultured in matrigel was used to determine the invasive capacity of indicated cells. **d** Statistic analysis showed the percentage of colonies with invasive structures

We further investigated the role of HOMER3 in the aggressiveness of breast cancer cells. As HOMER3 is substantially increased in TNBC, we first tested the effects by silencing the endogenous HOMER3 in human TNBC cell lines SUM159PT and MDA-MB-231, as well as a metastatic mouse TNBC cell line 4T1 (Additional file 4: Fig. 4A). As shown in Fig. 5, EGF treatment promotes the invasive capacities of TNBC cells. Remarkably, downregulation of HOMER3 inhibited TNBC cells to invade and form outward projections in matrigel (Fig. 5a–d). Notably, reintroduction of full-length HOMER3 rescued the aggressiveness of HOMER3-silencing TNBC cells, but the truncated EVH1 or CC domain had no effects (Fig. 5a–d). These findings suggest that HOMER3 is essential for the EGF-induced aggressive capacities in TNBC.

Conversely, the role of HOMER3 was examined in non-TNBC cells via exogenous overexpression. As expected, ectopic expression of HOMER3 robustly increased EGF-induced Tyr phosphorylation and activation of β-Catenin in MCF-7 and T47D cells (Additional file 4: Fig. 4b–d). Moreover, overexpression of HOMER3 rendered MCF-7 and T47D cells to invade and form outward projections in matrigel, while the control cells were immotile and displayed spheroid morphologies Additional file 44: Fig. 4E-G). These results further suggest that HOMER3 endows non-TNBC cells with aggressiveness.

Notably, silencing of c-Src abrogated HOMER3-mediated cellular invasion in MCF-7 and T47D cells, suggesting that expression of the upstream c-Src kinase is required for the pro-metastatic function of HOMER3 (Additional file 4: Fig. 4E-G). We further investigated whether the c-Src-P307L mutant, which lost the capacity to interact with HOMER3, affect EGF-stimulated β-Catenin phosphorylation and cell invasiveness. Of note, ectopic expression of c-Src-P307L showed no significant effects on β-catenin Tyr phosphorylation, p-β-Catenin-Y333 expression, and cell invasive capacity, with or without EGF stimulation in both TNBC MDA-MB-231 and non-TNBC MCF-7 cells (Additional file 5: Fig. 5A-C). These findings further reveal that c-Src expression, as well as its interaction with HOMER3, is indispensable for HOMER3 to facilitate EGF-mediated β-Catenin phosphorylation and cell aggressiveness.

### Silencing of HOMER3 inhibits cancer metastasis in TNBC

**Fig. 6:**
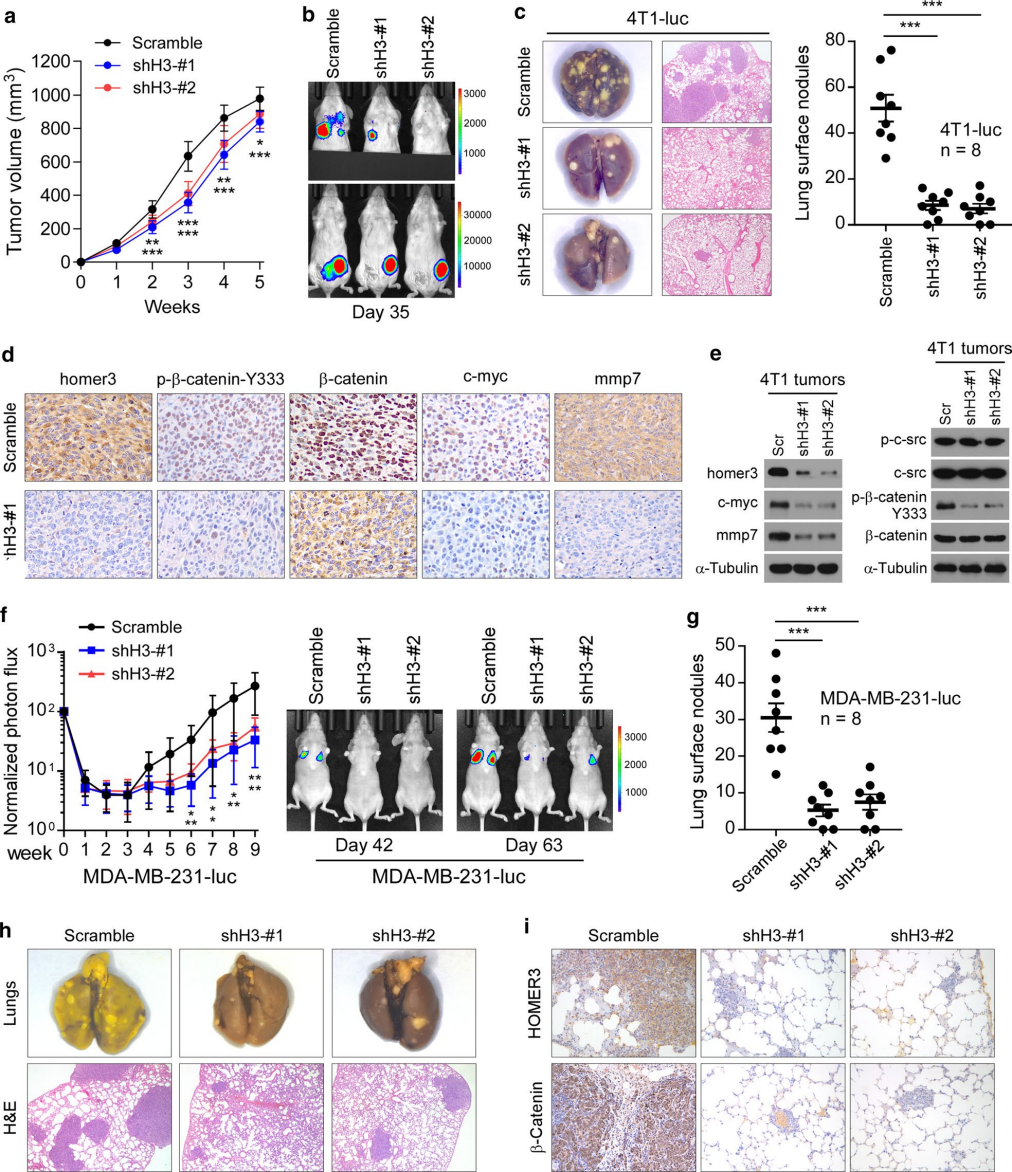
Silencing of HOMER3 inhibits cancer metastasis in TNBC. **a** Control and HOMER3 silencing 4T1-luc cells (2 × 10^5^, *n* = 8/group) that stably expressing firefly luciferase gene were orthotopically injected in BALB/c mice. Tumor volumes were calculated on indicated days. **b** Bioluminescence imaging of spontaneous metastasis on day 35. Upper panel, bioluminescence imaging of lung metastasis was photographed after blocking the orthotopic tumor signals. **c** Representative bright-field imaging and H&E confirmation of lung metastases. The number of visible surface lesions were shown as mean ± SEM. **d** IHC staining of HOMER3, p-β-Catenin-Y333, β-Catenin, c-Myc and MMP7 in 4T1 tumors. **e** Western blot analysis of indicated proteins in control and HOMER3-silencing 4T1 tumors. **f** Lung colonization model of MDA-MB-231-luc cells. 1 × 10^6^ control or HOMER3-silencing MDA-MB-231-luc cells were injected into BALB/c nude mice (*n* = 8/group) via lateral tail veins. Lung metastasis burden of animals was monitored weekly using bioluminescent imaging (BLI). Representative BLI images of mice on day 42 and 63 were shown. **g** Mice were euthanized 9 weeks after inoculation, and lung metastatic nodules were counted. **h** Lung metastases were confirmed by H&E staining. **i** IHC staining of HOMER3 and β-Catenin in lung metastases. ***P < 0.001

Furthermore, we used in vivo tumor models to assess the potential of targeting HOMER3 in breast cancer metastasis suppression. Firstly, the role of HOMER3 silencing was tested by a mouse 4T1 spontaneous metastasis model. The 4T1-luc cells (2 × 10^5^) with or without HOMER3 downregulation were implanted into the mammary fat pads of BALB/c female mice. Knockdown of HOMER3 showed an approximate 20% repression on the growth of 4T1 tumors (Fig. 6a). Indeed, the proliferation rate of TNBC cells could be slowed down by HOMER3 knockdown (Additional file 6: Fig. 6A). Strikingly, bioluminescence imaging indicated that silencing of HOMER3 strongly diminished lung metastasis of 4T1 cells (Fig. 6b). This was further confirmed by counting the visible metastatic lesions and H&E staining of lung tissues from sacrificed mice, showing about 80% reduction of metastases (Fig. 6c). Consistently, the metastatic index was significantly decreased in HOMER3-silencing 4T1 tumors (Additional file 6: Fig. 6B), further indicating a pro-metastatic role of HOMER3. Importantly, silencing of HOMER3 remarkably decreased the levels of p-β-Catenin-Y333, nuclear β-Catenin, c-Myc and MMP7 expression in 4T1 tumors without disruption of c-Src activity, further suggesting that HOMER3 mediated the c-Src to β-Catenin signal transduction (Fig. 6d, e).

Moreover, the role of HOMER3 silencing in metastatic outgrowth was tested via a human MDA-MB-231 lung colonization model. Luciferase-overexpressing MDA-MB-231 cells with or without HOMER3 knockdown were intravenously injected in immunodeficient BALB/c-nu mice. Strikingly, by tracking the luciferase signals, we observed that silencing of HOMER3 induced a significant delay in the metastatic outgrowth of lung burdens formed by MDA-MB-231 cells (Fig. 6f). Consistently, lung metastatic lesions formed by HOMER3 silencing MDA-MB-231 cells were significantly reduced (Fig. 6g, h). IHC staining on sections of lung metastases revealed that silencing of HOMER3 substantially reduced nuclear β-Catenin expression and p-β-Catenin-Y333, thus inhibiting the distant colonization of MDA-MB-231 cells (Fig. 6i and Additional file 6: Fig. 6C). Taken together, these results reveal that HOMER3 plays a crucial role in TNBC metastasis.

### Clinical relevance of HOMER3-induced β-Catenin activation in breast cancer

**Fig. 7:**
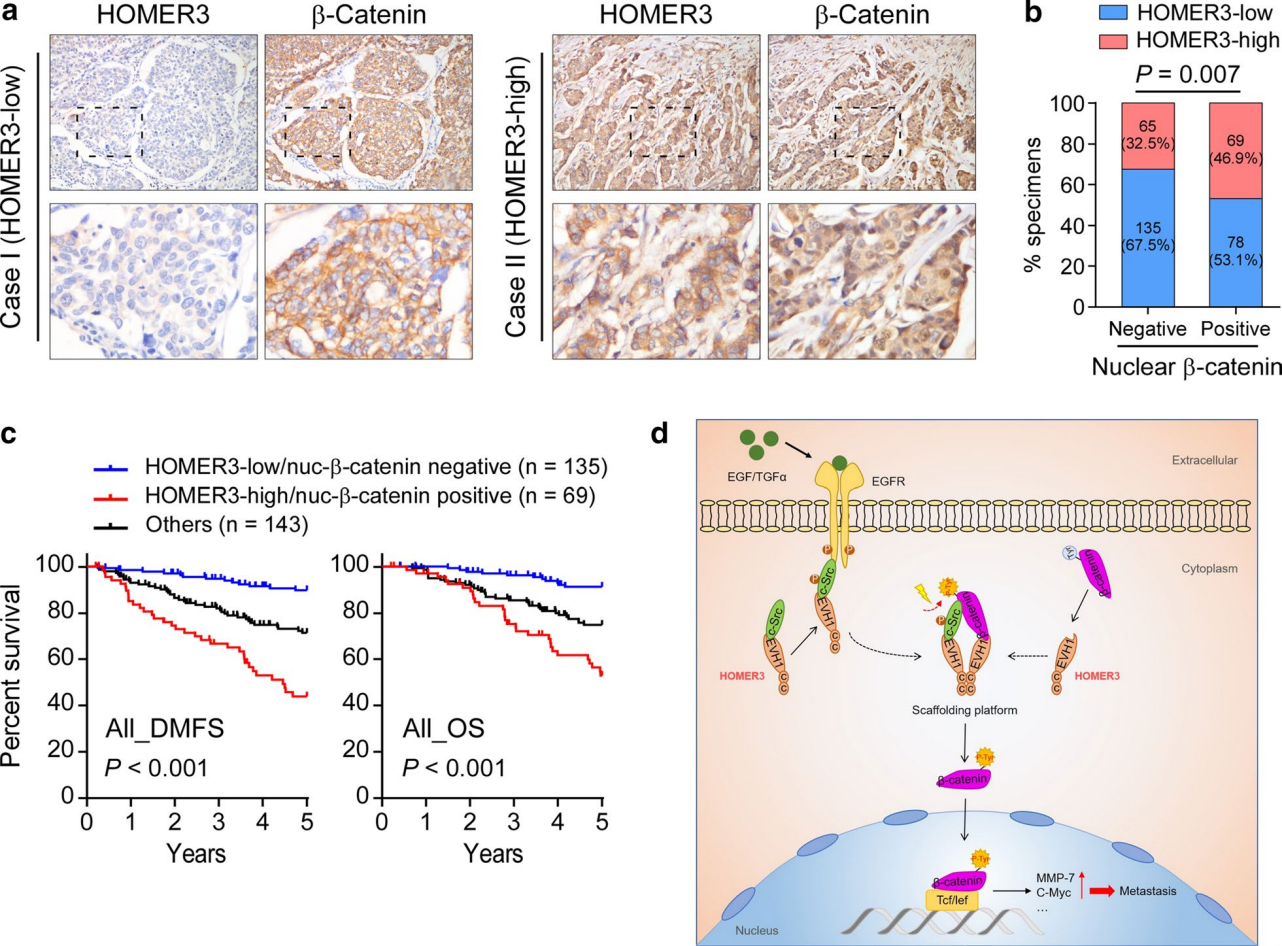
Clinical relevance of HOMER3-induced β-Catenin activation in breast cancer. **a, b** Representative images (**a**) and correlation analysis (**b**) of HOMER3 and nuclear β-Catenin staining in 347 breast cancer specimens. χ^2^ test was used. **c** The patient specimens were divided into three groups according to HOMER3 and nuclear β-Catenin expression. Kaplan–Meier survival curves showed that breast cancer patients with combined high HOMER3 and nuclear β-Catenin significantly suffered the worst DMFS and OS. **d** Study model: HOMER3 provides a scaffold platform to promote Tyr phosphorylation and activation of β-Catenin, leading to the malignant progression and poor clinical outcomes in human breast cancer

Finally, we evaluated the clinical relevance and significance of HOMER3/β-Catenin axis in breast cancer. Significantly, IHC staining and subsequent correlation analysis revealed that HOMER3 positively correlated with nuclear β-Catenin in the cohort of 347 breast cancer patient specimens (Fig. 7a, b). Importantly, Kaplan–Meier survival curves and log-rank tests revealed that patients with combined high HOMER3 expression and positive nuclear β-Catenin expression suffered poorest 5-year DMFS and OS in breast cancer patients (Fig. 7c). Taken together, our findings suggest that HOMER3 provides a scaffold platform to promote Tyr phosphorylation and activation of β-Catenin, leading to the malignant progression and poor clinical outcomes in human breast cancer (Fig. 7d).

## Discussion

Scaffolding proteins are required for the assembly of signal transduction complexes in response to extrinsic stimuli, such as growth factors, hormones and extracellular matrix components [31, 32]. Given their ability to integrate and coordinate multiple signaling events, HOMER scaffolding proteins have emerged as crucial players in the control of cell proliferation, survival and differentiation [20, 22, 33]. More importantly, it has been found that alterations of their expression levels result in aberrant signaling cascades, which promotes the development and progression of human diseases [23–25, 34]. In this study, we find that HOMER3 uses its EVH1 domain to recognize c-Src and β-Catenin, thus providing a scaffolding platform to increase the efficiency of c-Src-mediated β-Catenin tyrosine phosphorylation and activation under growth factors. These findings uncover a novel role of HOMER3 in the crosstalk between growth factor receptor signal transduction and canonical Wnt pathway, and suggest an oncogenic role of HOMER3 in cancer metastasis.

Constitutive activation of β-Catenin has been proved to contribute to malignant progression of TNBC [8, 9]. However, comprehensive genomic analysis revealed that activating mutations of certain Wnt/β-Catenin pathway components such as APC and β-Catenin were rarely observed in breast cancer, indicating that alternative mechanisms, probably Wnt-independent ways, exist to activate β-Catenin. Notably, β-Catenin could be phosphorylated on tyrosine residues by growth factors for excessive nuclear translocation and transcriptional activation of targeted genes in malignant cancers [12–14]. Nevertheless, whether and how the aggressive traits of TNBC cells are regulated by tyrosine phosphorylation-induced activation of β-Catenin remain unclear. Herein, we showed that the levels of β-Catenin tyrosine phosphorylation were substantially increased in TNBC compared with non-TNBC cells. We later found that growth factor stimulated β-Catenin tyrosine phosphorylation was accelerated by HOMER3 overexpression but impaired by silencing of HOMER3. HOMER3 promotes tumor aggressiveness and metastasis of TNBC cells via facilitating β-Catenin tyrosine phosphorylation. Thus, these findings provide a novel Wnt-independent mechanism for β-Catenin activation in TNBC, and suggest HOMER3 as a targeting vulnerability of β-Catenin signaling.

Undeniably, molecular-targeted therapy is integral for modern breast cancer treatment. Effective targeting therapy could transform deadly metastatic breast cancer into a controllable chronic disease to some extent. However, considering that TNBC tumors lack expression of hormone and Her2 receptors, and these patients cannot benefit from endocrine therapy or targeted medicine such as trastuzumab [35]. Although, conventional radiotherapy and chemotherapy have demonstrated efficacy in the treatment of TNBC, these treatments are either transient or effective in a limited set of patients due to frequent and early occurrence of metastasis [5]. In this study, our results show that HOMER3 is selectively overexpressed in TNBC and correlate earlier tumor metastasis and shorter patient survival. Importantly, silencing of HOMER3 robustly inhibits the invasion and metastatic outgrowth of TNBC cells, suggesting that HOMER3 might be a potential therapeutic target against TNBC.

Notably, a recent report indicates that genomic amplification contributes to HOMER3 overexpression in esophageal squamous cell carcinoma [24, 36]. However, no significant copy number alteration of the HOMER3 genomic locus could be observed from the TCGA breast cancer dataset [11]. Interestingly, a research dedicated onto the spine and dendrite degeneration in spinocerebellar ataxia presented by Ruegsegger et al. identified HOMER3 as a downstream target of mTORC1 since ablation of mTORC1 signaling leads to reduced HOMER3 levels in cerebellar Purkinje cells and vice versa [37]. However, we here did not observe any significant effects of EGF/TGF-α on the expression of HOMER3, suggesting that HOMER3 itself is not a direct downstream gene of EGFR signaling. Future investigation of the mechanism for the specific upregulation of HOMER3 in TNBC might provide new clues for the targeting of HOMER3.

In summary, our study reveals that HOMER3 is a critical regulator in growth factor-induced β-Catenin activation and promotes metastasis in breast cancer. Understanding the precise role of HOMER3 in breast cancer pathogenesis and in the assembly of c-Src/β-Catenin complex promises to increase our knowledge of the biological basis of TNBC malignant progression and may also facilitate the development of new therapeutic strategies against TNBC.

## Conclusions

Triple-negative breast cancer (TNBC) is the most challenging subtype of human breast cancer, demanding new biomarkers and therapeutic targets. HOMER family proteins (HOMER1-3) are scaffolding proteins that regulate the assembly of signal transduction complexes in response to extrinsic stimuli. However, the role of HOMER protein in breast cancer, especially the potential in TNBC metastasis remains unclear. In this study, we identified that HOMER3 was selectively overexpressed in TNBC and associated with poor prognosis. Intriguingly, HOMER3 was essential for growth factor-mediated but not canonical Wnt-induced β-Catenin activation. HOMER3 used its EVH1 domain to recognize and interact with both c-Src and β-Catenin, and utilized the coiled-coil domain to provide a multimeric scaffolding platform to facilitate c-Src-induced β-Catenin tyrosine phosphorylation under growth factor stimulation. Importantly, silencing of HOMER3 robustly inhibited TNBC metastasis in vivo. These findings uncover a novel role of HOMER3 in growth factor-mediated constitutive activation β-Catenin in TNBC and suggest that HOMER3 might be a targetable vulnerability of TNBC.

## Supplementary Information

**Additional file 1.** **Figure 1**. (A) Expression levels of HOMER family genes in normal, non-TNBC and TNBC samples from The Cancer Genome Atlas (TCGA) dataset. (B) Similar to (A), analysis was compared in different molecular subtypes. (C) Real-time PCR analysis of HOMER family genes in 5 normal breast tissues, 10 non-TNBC and 10 TNBC tissues. (D) The prognostic values of HOMER3 in DMFS, relapse-free survival (RFS), and OS of breast cancer patients were further assessed by a public Kaplan-Meier Plotter program (http://kmplot.com/analysis). All settings were default except the following ones: probe (204647_at), survival (DMFS, RFS or OS), and auto select best cutoff (on).

**Additional file 2.** **Figure 2.** (A) Normalized luciferase activities of specific TOP-Flash over non-specific FOP-Flash relative renilla luciferase units (RLU) in MDA-MB-231 and SUM159PT cells cultured with 1% or 10% Fetal Bovine Serum (FBS). ns, not significant. (B) Total and phosphorylation levels of EGFR and c-Src in MDA-MB-231, SUM159PT, MCF-7 and T47D cells treated with or without EGF. (C) Western blot analysis of p-EGFR, EGFR, p-c-Src, c-Src in control and HOMER-3 silencing MDA-MB-231 cells treated with PBS, EGF, or TGFα. (D) Real-time PCR analysis of HOMER3 in MDA-MB-231 cells treated with PBS, EGF, or TGFα.

**Additional file 3.** **Figure 3**. (A) Western blot analysis of HOMER3, c-Src and β-Catenin in indicated cells. α-Tubulin was used as a loading control. (B) Normalized luciferase activities of specific TOP-Flash over non-specific FOP-Flash relative renilla luciferase units (RLU) in control or HOMER3-overexpressing TNBC cells with or without c-Src silencing.

**Additional file 4.** **Figure 4.** (A) Western blot analysis of HOMER3 in MDA-MB-231, SUM159PT and 4T1 cells that stably transduced with HOMER3 shRNA#1 or shRNA#2. (B) Tyr phosphorylation levels of β-Catenin in indicated cells. (C) Western blot analysis in control and HOMER3-overexpressing MCF-7 and T47D cells with or without EGF treatment. (D) Normalized luciferase activities of specific TOP-Flash over non-specific FOP-Flash relative renilla luciferase units (RLU) in MCF-7 and T47D with PBS or EGF treatment. (E) Knockdown of c-Src in control or HOMER3 overexpressing MCF-7 and T47D cells was validated by western blot analysis. (F) Representative images and quantification of invading MCF-7 and T47D cells in the transwell matrix penetration assays. (G) 3-D spheroid cultured in matrigel was used to determine the invasive capacity of indicated cells.

S**Additional file 5.** **Figure 5.** (A) Western blot analysis of indicated proteins in control or c-Src-P307L overexpressing MDA-MB-231 and MCF-7 cells with or without EGF treatment. (B) Tyr phosphorylation levels of β-Catenin in indicated cells. (C) Representative images and quantification of invading MDA-MB-231 and MCF-7 in the transwell matrix penetration assays.

**Additional file 6.** **Figure 6.** (A) The proliferation rate of control or HOMER3 silencing 4T1, MDA-MB-231 and SUM159PT cells was examined by MTT assay. (B) The metastatic index (ratio of nodule number/tumor volume) in each group was calculated by the ratio of nodule number to tumor volume. (C) IHC staining of p-β-Catenin-Y333 in MDA-MB-231 lung metastases.

**Additional file 7.** Supplementary materials, methods, and tables.

## Data Availability

Not applicable.

## Abbreviations

TNBC: Triple-negative breast cancer
TCF: T-cell factor
LEF: Lymphoid enhancer factor
EVH1: The enabled/vasodilator-stimulated phosphoprotein homology 1
PIKE-L: Phosphoinositide 3 kinase enhancer-long isoform
mGluRs: Group 1 metabotropic glutamate receptors
IP3R: IP3 receptor
IHC: Immunohistochemistry
TCGA: The cancer genome atlas
Tyr: Tyrosine
IP: Immunoprecipitation
IF: Immunofluorescence
CC: Coiled-coil
