# Supplementary figures and images for: HOMER3 facilitates growth factor-mediated β-Catenin tyrosine phosphorylation and activation to promote metastasis in triple negative breast cancer
Source: J Hematol Oncol. 2021 Jan 6;14:6. doi: 10.1186/s13045-020-01021-x (PMC7788750; doi:10.1186/s13045-020-01021-x)

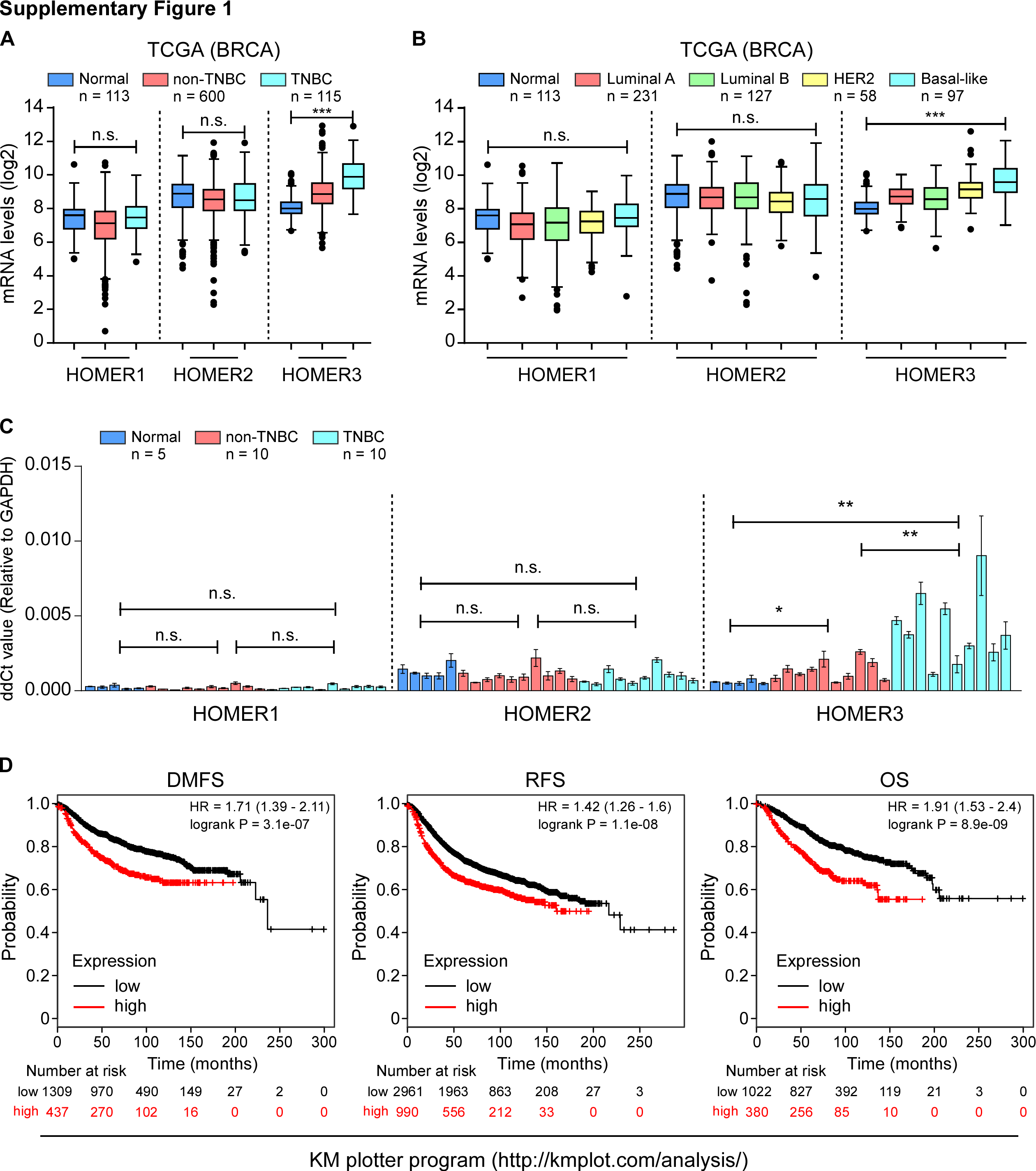

Supplement: Supplementary file 1 — Additional file 1. Figure 1. (A) Expression levels of HOMER family genes in normal, non-TNBC and TNBC samples from The Cancer Genome Atlas (TCGA) dataset. (B) Similar to (A), analysis was compared in different molecular subtypes. (C) Real-time PCR analysis of HOMER family genes in 5 normal breast tissues, 10 non-TNBC and 10 TNBC tissues. (D) The prognostic values of HOMER3 in DMFS, relapse-free survival (RFS), and OS of breast cancer patients were further assessed by a public Kaplan-Meier Plotter program (http://kmplot.com/analysis). All settings were default except the following ones: probe (204647_at), survival (DMFS, RFS or OS), and auto select best cutoff (on). [file 13045_2020_1021_MOESM1_ESM.tif]

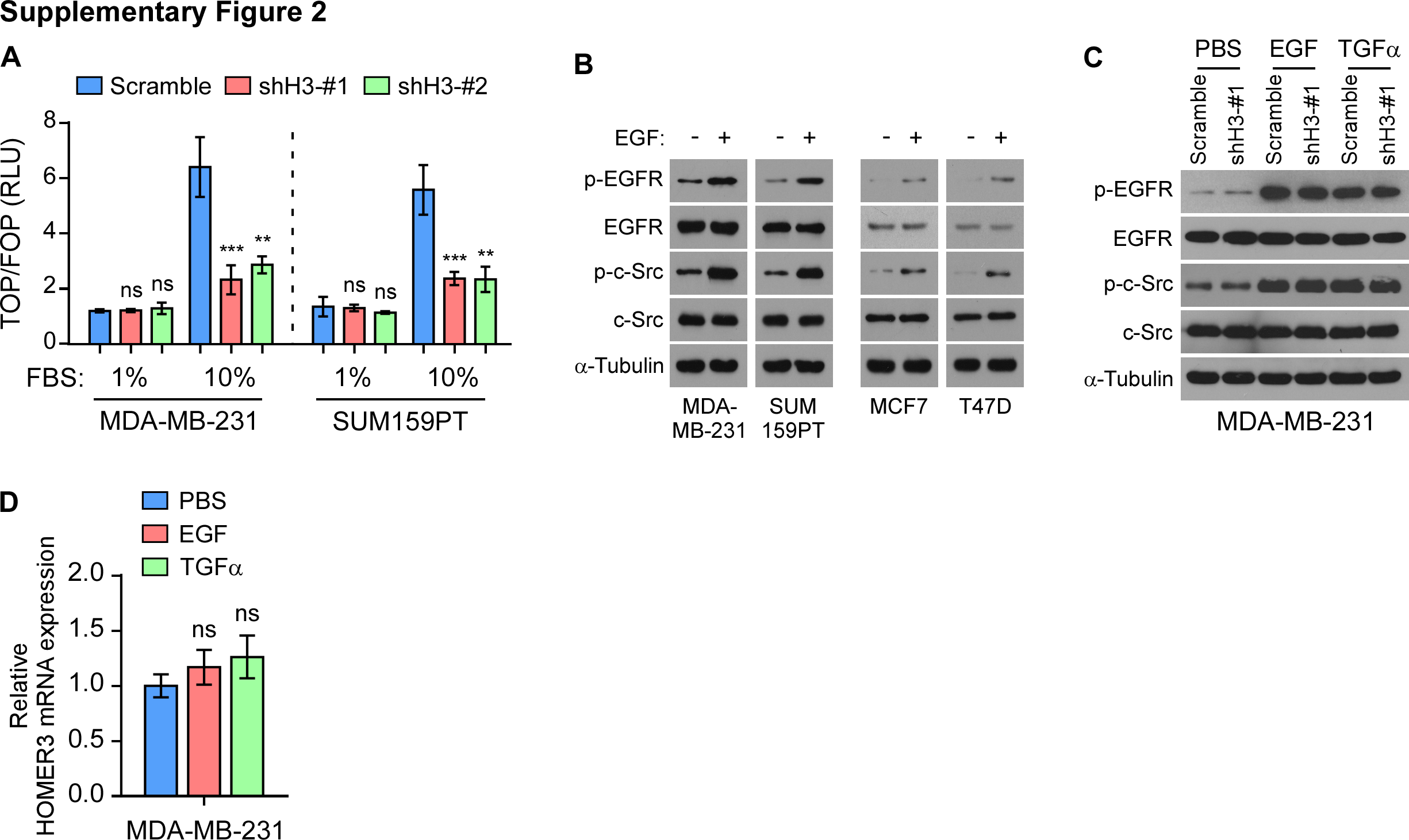

Supplement: Supplementary file 2 — Additional file 2. Figure 2. (A) Normalized luciferase activities of specific TOP-Flash over non-specific FOP-Flash relative renilla luciferase units (RLU) in MDA-MB-231 and SUM159PT cells cultured with 1% or 10% Fetal Bovine Serum (FBS). ns, not significant. (B) Total and phosphorylation levels of EGFR and c-Src in MDA-MB-231, SUM159PT, MCF-7 and T47D cells treated with or without EGF. (C) Western blot analysis of p-EGFR, EGFR, p-c-Src, c-Src in control and HOMER-3 silencing MDA-MB-231 cells treated with PBS, EGF, or TGFα. (D) Real-time PCR analysis of HOMER3 in MDA-MB-231 cells treated with PBS, EGF, or TGFα. [file 13045_2020_1021_MOESM2_ESM.tif]

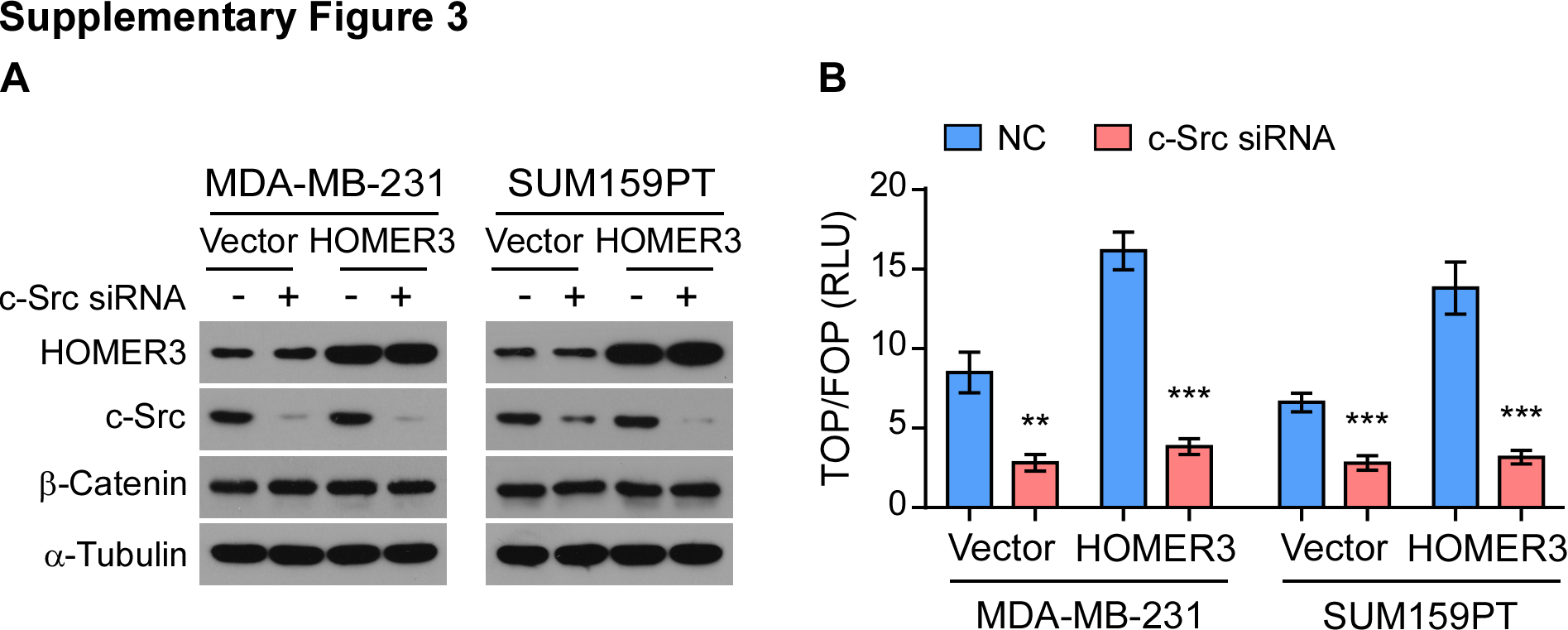

Supplement: Supplementary file 3 — Additional file 3. Figure 3. (A) Western blot analysis of HOMER3, c-Src and β-Catenin in indicated cells. α-Tubulin was used as a loading control. (B) Normalized luciferase activities of specific TOP-Flash over non-specific FOP-Flash relative renilla luciferase units (RLU) in control or HOMER3-overexpressing TNBC cells with or without c-Src silencing. [file 13045_2020_1021_MOESM3_ESM.tif]

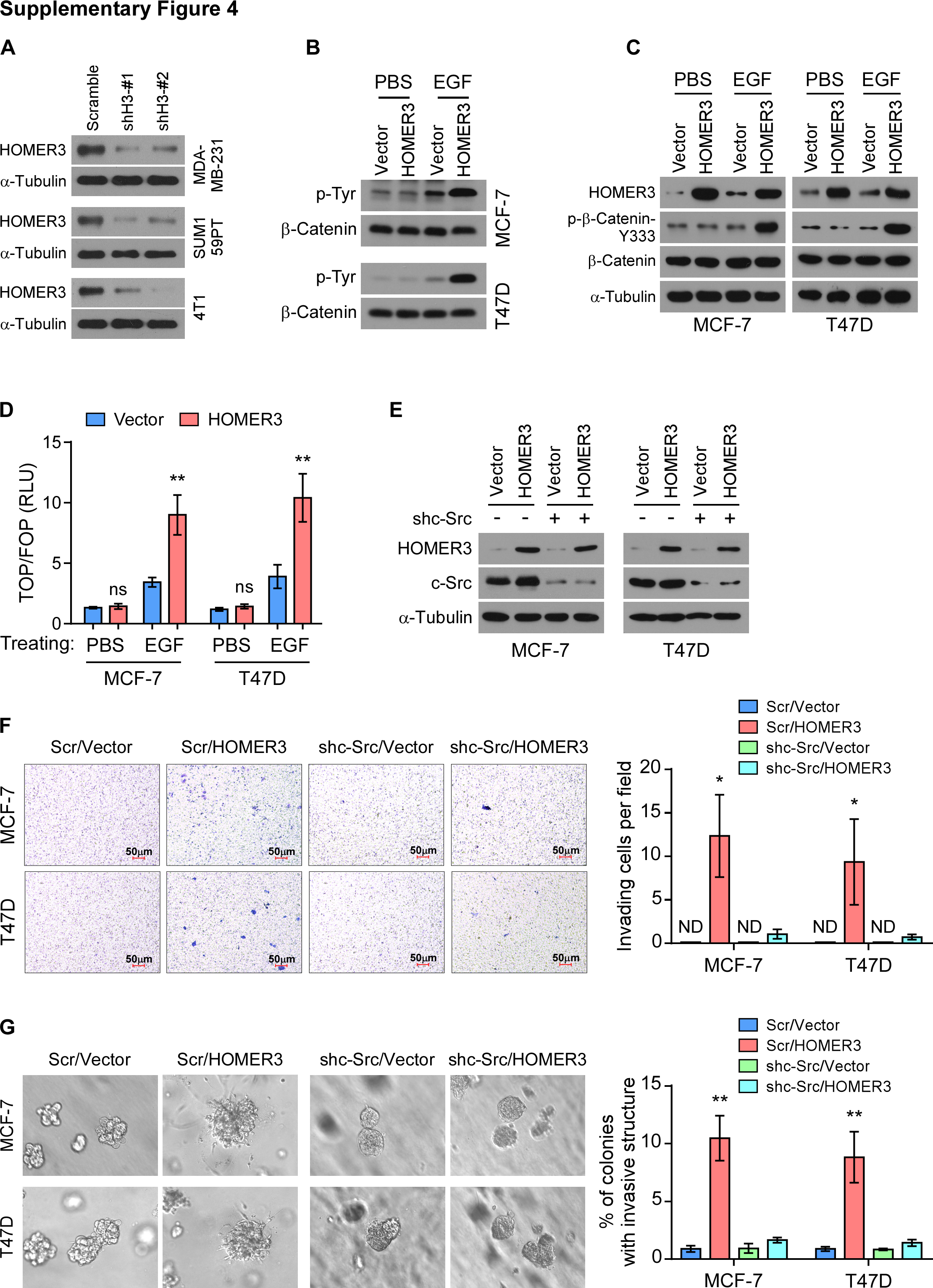

Supplement: Supplementary file 4 — Additional file 4. Figure 4. (A) Western blot analysis of HOMER3 in MDA-MB-231, SUM159PT and 4T1 cells that stably transduced with HOMER3 shRNA#1 or shRNA#2. (B) Tyr phosphorylation levels of β-Catenin in indicated cells. (C) Western blot analysis in control and HOMER3-overexpressing MCF-7 and T47D cells with or without EGF treatment. (D) Normalized luciferase activities of specific TOP-Flash over non-specific FOP-Flash relative renilla luciferase units (RLU) in MCF-7 and T47D with PBS or EGF treatment. (E) Knockdown of c-Src in control or HOMER3 overexpressing MCF-7 and T47D cells was validated by western blot analysis. (F) Representative images and quantification of invading MCF-7 and T47D cells in the transwell matrix penetration assays. (G) 3-D spheroid cultured in matrigel was used to determine the invasive capacity of indicated cells. [file 13045_2020_1021_MOESM4_ESM.tif]

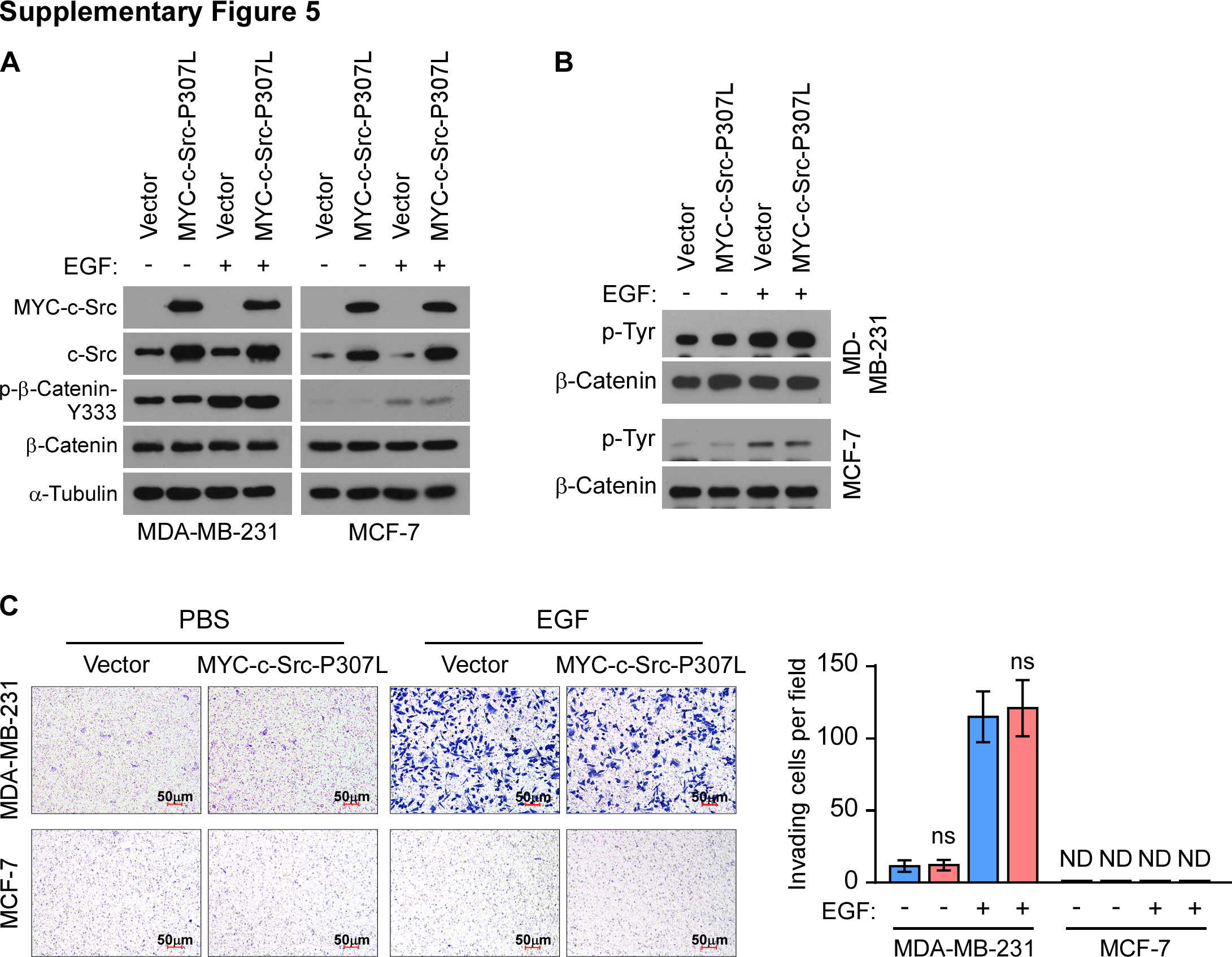

Supplement: Supplementary file 5 — SAdditional file 5. Figure 5. (A) Western blot analysis of indicated proteins in control or c-Src-P307L overexpressing MDA-MB-231 and MCF-7 cells with or without EGF treatment. (B) Tyr phosphorylation levels of β-Catenin in indicated cells. (C) Representative images and quantification of invading MDA-MB-231 and MCF-7 in the transwell matrix penetration assays. [file 13045_2020_1021_MOESM5_ESM.tif]

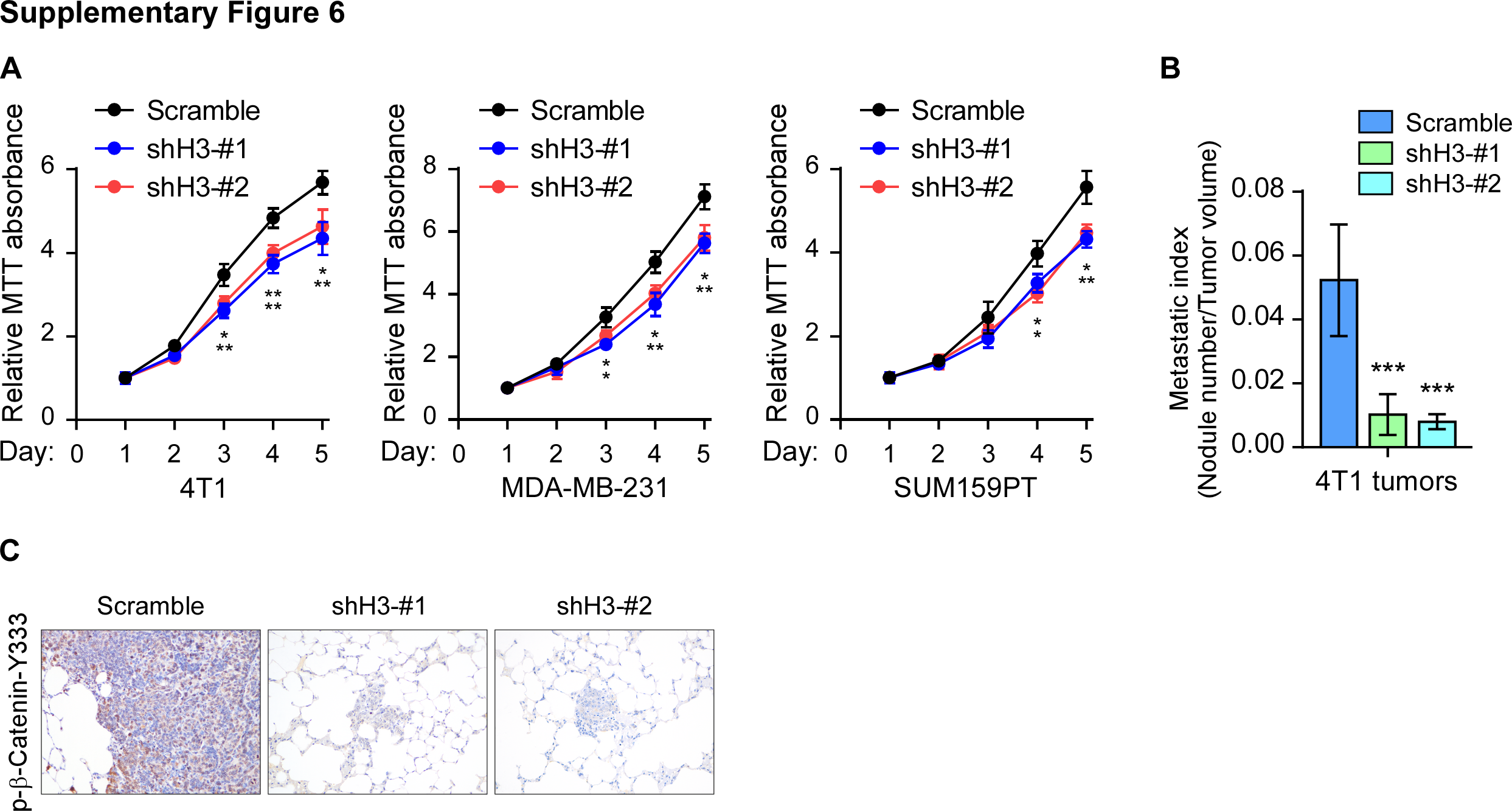

Supplement: Supplementary file 6 — Additional file 6. Figure 6. (A) The proliferation rate of control or HOMER3 silencing 4T1, MDA-MB-231 and SUM159PT cells was examined by MTT assay. (B) The metastatic index (ratio of nodule number/tumor volume) in each group was calculated by the ratio of nodule number to tumor volume. (C) IHC staining of p-β-Catenin-Y333 in MDA-MB-231 lung metastases. [file 13045_2020_1021_MOESM6_ESM.tif]
