# Supplementary material for: HOMER3 facilitates growth factor-mediated β-Catenin tyrosine phosphorylation and activation to promote metastasis in triple negative breast cancer
Source: J Hematol Oncol. 2021 Jan 6;14:6. doi: 10.1186/s13045-020-01021-x (PMC7788750; doi:10.1186/s13045-020-01021-x)
Supplement: Supplementary file 7 — Additional file 7. Supplementary materials, methods, and tables. [file 13045_2020_1021_MOESM7_ESM.docx]

**SUPPLEMENTARY INFORMATION**

**Supplementary Materials and Methods**

**Immunohistochemistry (IHC)**

In this study, IHC staining was done in 347 human breast cancer tissues. In brief, paraffin-embedded specimens were cut into 4-μm sections and baked at 65°C for 30 min. The sections were deparaffinized with xylenes and rehydrated. Sections were then submerged into EDTA antigenic retrieval buffer and microwaved for antigenic retrieval. Samples were treated with 3% hydrogen peroxide in methanol to quench the endogenous peroxidase activity, followed by incubation with 1% bovine serum albumin to block nonspecific binding, and then incubated with primary antibodies overnight at 4°C. After washing, the tissue sections were treated with biotinylated secondary antibody, followed by further incubation with streptavidin-horseradish peroxidase complex (Zsbio, BJ, China). Finally, the sections were immersed in 3-amino-9-ethyl carbazole and counterstained with 10% Mayer’s hematoxylin, dehydrated, and mounted in Crystal Mount.

Primary antibodies used in the IHC staining include anti-HOMER3 (Sigma-Aldrich, HPA040999, 1:1000), and anti-β-Catenin (Cell Signaling Technology, #8480, 1:100). The specificity of anti-HOMER3 antibody was validated. For negative controls, the primary antibody was replaced with normal rabbit serum by coincubation at 4°C overnight preceding the immunohistochemical staining procedure.

**Western blot analysis**

Western blot analyses were performed using primary antibodies including anti-HOMER3 (Sigma-Aldrich), anti-c-Src, anti-β-Catenin, anti-p-Tyr, anti-p-β-Catenin-S33/37/T41 (Cell Signaling Technology), anti-p-β-catenin-Y333 (Abcam) antibodies. The membranes were stripped and re-probed with anti-α-Tubulin as a loading control. P84 was used as a nuclear marker.

**DNA constructs and establishment of stable cell lines**

Full-length and truncated human HOMER3, c-Src and β-Catenin sequences were subcloned into pLVX-retro-hygro vector. To silence endogenous HOMER3, two short hairpin RNA (shRNA) oligonucleotides were cloned into pSuper-retro-neo vector. The targeting sequences of shRNAs were as followed: shH3-#1, 5’-CAAACCAAGGACCAGGAGATT-3’; shH3-#2, 5’-CGGCTAAAGAAGATGTTGTCT-3’. To re-expressed HOMER3 in shH3-#1 cells, HOMER3 constructs were edited with same sense mutations to prevent shH3-#1 mediated downregultion.

Stable cells were generated from cell pools by retroviral infection using the pLVX-retro-hygro for HOMER3 overexpression, and p-Super-retro-neo for HOMER3 silencing. Briefly, the retroviral vectors were co-transfected with packaging plasmid into 293T cells. Supernatant containing the virus were collected and viral infections were done serially for 3 days. Stable cell lines expressing HOMER3 were selected with 25 μg/ml hygromycin, and cells stably silenced by HOMER3-shRNAs were selected with 250 μg/ml G418 for 10 days.

**Transwell matrix invasion assay**

Cells (1 × 10^4^) suspended in serum free medium or EGF-supplemented medium (50μl) were plated into the upper chamber of polycarbonate transwell filters coated with Matrigel (BD Biosciences, San Jose, CA, USA), cultured at 37°C for 24 h, then the cells inside the upper chamber were removed with cotton swabs and the cells that had migrated to the bottom surface of the membrane where a 10% FBS attraction was prepared, were fixed in 1% paraformaldehyde, stained with crystal violet and counted in five random fields of view per well. The data was shown as mean + SD.

**3-D spheroid invasion assay**

Cells (1 × 10^4^) were trypsinized, suspended in medium with 2% Matrigel (BD Biosciences), and then seeded in 24-well plates. Two days after seeding, cells were replenished with 50μl serum-free or EGF-supplemented medium every other day. The invasive capacity of cells was calculated by the percentage of colonies with invasive structure. The data was shown as mean + SD.

**Immunofluorescence**

Cells (5×10^4^) were plated on coverslips. The cells were washed three times with PBS and treated with PBS containing 1% Triton X-100. Next, cells were stained with primary anti-β-Catenin antibody (Cell Signaling Technology, #8480, 1:100) for 2 hours at 4°C according to the manufacturer’s instructions. For the colonization staining of c-Src and HOMER3, cells were first stimulated with EGF for a short time, and then stained with anti-c-Src and anti-HOMER3 antibodies. After washing three times with PBS, the cells were incubated with rhodamine-conjugated goat anti-rabbit or anti-mouse antibody (Cell Signaling Technology, 1:100) at 37°C for 1 hour. Cells were counterstained with DAPI (Sigma-Aldrich) to visualize the nuclei. The percentage of membrane colonization of c-Src and HOMER3 was counted in five random fields.

**Gene expression profiling and analysis**

Analyses were performed using the TCGA data and Gene expression Omnibus (GEO) publically available human breast cancer datasets. Information of clinical characters including ER, PR and HER2 expression status, TNBC or non-TNBC status, as well as the molecular subtypes of samples were obtained according to their respective clinical information files. TCGA data include 113 normal, 600 non-TNBC, 115 TNBC, 231 luminal A, 127 luminal B, 58 Her2, 97 basal-like, and 8 normal-like samples. GSEA was performed using GSEA program (http://www.broadinstitute.org/gsea/).

**Supplementary figure legends**

**Supplementary Figure 1.** **(A)** Expression levels of HOMER family genes in normal, non-TNBC and TNBC samples from The Cancer Genome Atlas (TCGA) dataset. **(B)** Similar to (A), analysis was compared in different molecular subtypes. **(C)** Real-time PCR analysis of HOMER family genes in 5 normal breast tissues, 10 non-TNBC and 10 TNBC tissues. **(D)** The prognostic values of HOMER3 in DMFS, relapse-free survival (RFS), and OS of breast cancer patients were further assessed by a public Kaplan-Meier Plotter program (http://kmplot.com/analysis). All settings were default except the following ones: probe (204647_at), survival (DMFS, RFS or OS), and auto select best cutoff (on).

**Supplementary Figure 2.** **(A)** Normalized luciferase activities of specific TOP-Flash over non-specific FOP-Flash relative renilla luciferase units (RLU) in MDA-MB-231 and SUM159PT cells cultured with 1% or 10% Fetal Bovine Serum (FBS). ns, not significant. **(B)** Total and phosphorylation levels of EGFR and c-Src in MDA-MB-231, SUM159PT, MCF-7 and T47D cells treated with or without EGF. **(C)** Western blot analysis of p-EGFR, EGFR, p-c-Src, c-Src in control and HOMER-3 silencing MDA-MB-231 cells treated with PBS, EGF, or TGFα. **(D)** Real-time PCR analysis of HOMER3 in MDA-MB-231 cells treated with PBS, EGF, or TGFα.

**Supplementary Figure 3.** **(A)** Western blot analysis of HOMER3, c-Src and β-Catenin in indicated cells. α-Tubulin was used as a loading control. **(B)** Normalized luciferase activities of specific TOP-Flash over non-specific FOP-Flash relative renilla luciferase units (RLU) in control or HOMER3-overexpressing TNBC cells with or without c-Src silencing.

**Supplementary Figure 4.** **(A)** Western blot analysis of HOMER3 in MDA-MB-231, SUM159PT and 4T1 cells that stably transduced with HOMER3 shRNA#1 or shRNA#2. **(B)** Tyr phosphorylation levels of β-Catenin in indicated cells. **(C)** Western blot analysis in control and HOMER3-overexpressing MCF-7 and T47D cells with or without EGF treatment. **(D)** Normalized luciferase activities of specific TOP-Flash over non-specific FOP-Flash relative renilla luciferase units (RLU) in MCF-7 and T47D with PBS or EGF treatment.  **(E)** Knockdown of c-Src in control or HOMER3 overexpressing MCF-7 and T47D cells was validated by western blot analysis. **(F)** Representative images and quantification of invading MCF-7 and T47D cells in the transwell matrix penetration assays. **(G)** 3-D spheroid cultured in matrigel was used to determine the invasive capacity of indicated cells.

**Supplementary Figure 5.** **(A)** Western blot analysis of indicated proteins in control or c-Src-P307L overexpressing MDA-MB-231 and MCF-7 cells with or without EGF treatment. **(B)** Tyr phosphorylation levels of β-Catenin in indicated cells. **(C)** Representative images and quantification of invading MDA-MB-231 and MCF-7 in the transwell matrix penetration assays.

**Supplementary Figure 6.** **(A)** The proliferation rate of control or HOMER3 silencing 4T1, MDA-MB-231 and SUM159PT cells was examined by MTT assay. **(B)** The metastatic index (ratio of nodule number/tumor volume) in each group was calculated by the ratio of nodule number to tumor volume. **(C)** IHC staining of p-β-Catenin-Y333 in MDA-MB-231 lung metastases.

**Supplementary Tables**

**Supplementary Table S1. Clinicopathological characteristics of 347 breast cancer specimens**

| Parameters | Number of cases (%) |
| --- | --- |
| **Gender** |  |
| Female | 347 (100.0) |
| **Age (years)** |  |
| < 50 | 168 (48.41) |
| ≥ 50 | 179 (51.59) |
| **Pathological type** |  |
| Invasive ductal carcinoma | 338 (97.41) |
| Others | 9 (2.59) |
| **T classification** |  |
| T_1_ | 104 (29.97) |
| T_2_ | 202 (58.21) |
| T_3_ | 22 (6.34) |
| T_4_ | 19 (5.48) |
| **N classification** |  |
| N_0_ | 151 (43.52) |
| N_1_ | 105 (30.26) |
| N_2_ | 47 (13.54) |
| N_3_ | 44 (12.68) |
| **Clinical stage** |  |
| I | 69 (19.88) |
| II | 174 (50.14) |
| III | 104 (29.98) |
| **Pathological grade** |  |
| G1 | 14 (4.03) |
| G2 | 213 (61.38) |
| G3 | 120 (34.59) |
| **ER expression** |  |
| Negative | 153 (44.09) |
| Positive | 194 (55.91) |
| **PR expression** |  |
| Negative | 162 (46.69) |
| Positive | 185 (53.31) |
| **HER2 expression** |  |
| Negative | 262 (75.50) |
| Positive | 85 (24.50) |
| **TNBC** |  |
| No | 256 (73.78) |
| Yes | 91 (26.22) |
| **Ki67 expression** |  |
| < 15% | 129 (37.18) |
| > 15% | 218 (62.82) |
| **5-year distant metastasis status** |  |
| No | 261 (75.22) |
| Yes | 86 (24.78) |
| **5-year vital status** |  |
| Alive | 274 (78.96) |
| Dead | 73 (21.04) |
| **HOMER3 expression** |  |
| Low | 213 (61.38) |
| High | 134 (38.62) |
| **Nuclear β-catenin expression** |  |
| Negative | 200 (57.64) |
| Positive | 147 (42.36) |

**Supplementary Table S2. Correlation between HOMER3 and clinicopathological characteristics of breast cancer patients**

|  | **HOMER3 expression** | |  |
| --- | --- | --- | --- |
| Characteristics | Low,  no. cases | High,  no. cases | *P* values |
| **Age (years)** |  |  |  |
| < 50 | 100 | 68 | 0.510 |
| ≥ 50 | 113 | 66 |  |
| **T** **stage** |  |  |  |
| T_1_ | 82 | 22 | < 0.001 |
| T_2_ – T_4_ | 131 | 112 |  |
| **N stage** |  |  |  |
| N_0_ | 113 | 38 | < 0.001 |
| N_1_ – N_3_ | 100 | 96 |  |
| **Clinical stage** |  |  |  |
| I – II | 172 | 71 | < 0.001 |
| III | 41 | 63 |  |
| **Pathological grade** |  |  |  |
| G_1_ – G_2_ | 144 | 83 | 0.298 |
| G_3_ | 69 | 51 |  |
| **TNBC** |  |  |  |
| No | 168 | 88 | 0.008 |
| Yes | 45 | 46 |  |
| **Ki67 expression** |  |  |  |
| < 15% | 85 | 44 | 0.210 |
| > 15% | 128 | 90 |  |
| **5-year distant metastasis status** |  |  |  |
| No | 181 | 80 | < 0.001 |
| Yes | 32 | 54 |  |
| **5-year vital status** |  |  |  |
| Alive | 184 | 90 | < 0.001 |
| Dead | 29 | 44 |  |
| **Nuclear β-catenin expression** |  |  |  |
| Negative | 135 | 65 | 0.007 |
| Positive | 78 | 69 |  |
